# The color-tuning mechanism in multi-resonance thermally activated delayed fluorescence emitters: site effects in peripheral modification and skeleton fusion†

**DOI:** 10.1039/d5sc01751c

**Published:** 2025-05-20

**Authors:** Zicong Situ, Xingqing Li, Shengsheng Wei, Xiang Wang, Yang Li, Yan Wan, Lian Duan, Andong Xia, Zhuoran Kuang

**Affiliations:** a State Key Laboratory of Information Photonic and Optical Communications, School of Science, Beijing University of Posts and Telecommunications (BUPT) Beijing 100876 P. R. China andongxia@bupt.edu.cn kuang@bupt.edu.cn; b Key Laboratory of Organic Optoelectronics & Molecular Engineering of Ministry of Education, Department of Chemistry, Tsinghua University Beijing 100084 P. R. China duanl@mail.tsinghua.edu.cn; c College of Chemistry, Beijing Normal University Beijing 100875 P. R. China

## Abstract

Ultra-narrowband, highly modifiable multi-resonance thermally activated delayed fluorescence (MR-TADF) materials are critical for high-performance, wide-color-gamut display applications, yet balancing broad color tunability while maintaining high color purity remains challenging. We present a proof-of-concept study into 7,10-di-*tert*-butyl-5-mesityl-5*H*-benzo[5,6][1,4]azaborinin-o[3,2,1 *jk*]carbazole (BIC) derivatives with a B/N MR skeleton to elucidate the site effects of peripheral modification and skeleton fusion on MR-TADF properties. Incorporating electron donating groups (EDGs) in a *meta*-(N–π–N) configuration forms D–void–A motifs, which retain or even enhance the MR characteristics, achieving a blue-shifted emission. In contrast, EDG modifications in a *para*-(N–π–N) configuration result in conjugative D–π–A motifs, leading to a red-shift in emission. Rigid skeleton fusion in an extended D–π–A motif compensates for the potential increase in vibronic coupling, which typically arises from a decline in MR properties. Furthermore, the centrosymmetric fused skeleton suppresses the excited-state structural relaxation in dielectric environments, achieving narrowband green emission (528 nm, 30 nm or 929 cm^−1^ in emission FWHM). This work elucidates the intrinsic mechanism of the widely applicable site effects in regulating MR characteristics and color-tuning *via* peripheral modification and skeleton fusion, providing a theoretical foundation for the design of ultrapure long-wavelength emission MR-TADF materials.

## Abstract 2

B/N-skeleton compounds exhibit MR-TADF properties. In skeleton modifications and fusions, “void sites” and “conjugative sites” balance the inductive and conjugative effects, enabling precise color-tuning while suppressing emission broadening.

## Graphical abstract

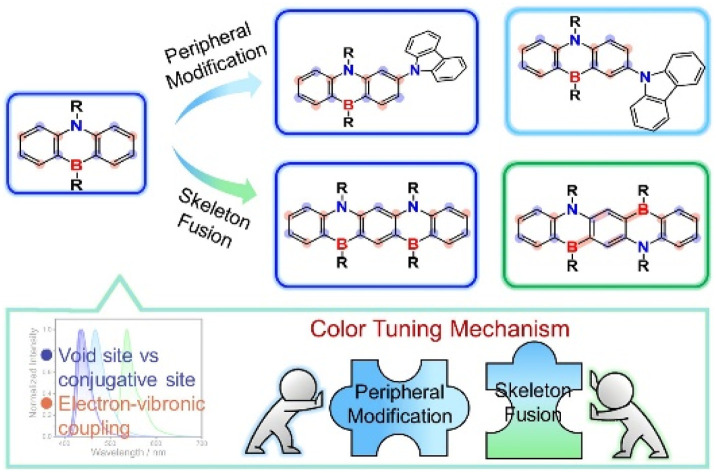

## Introduction

**Fig. 1:**
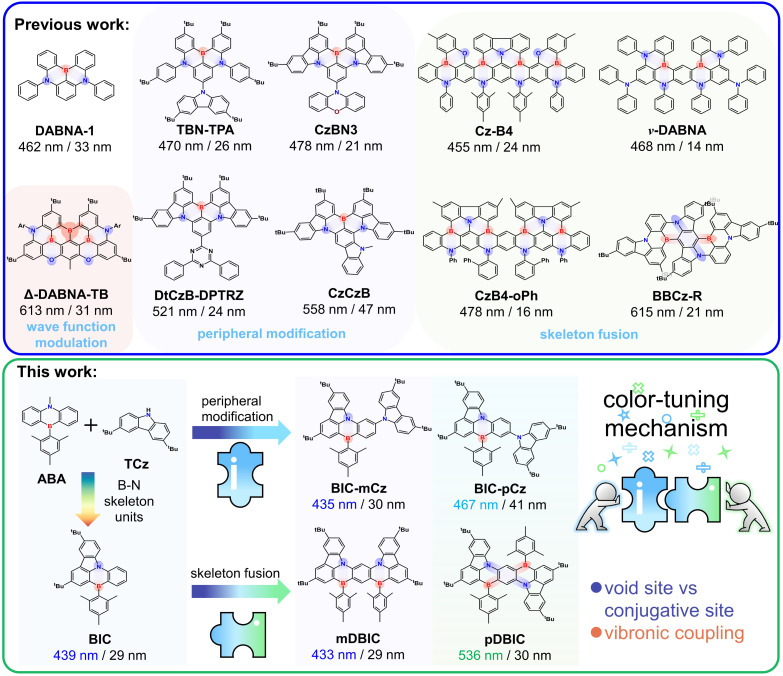
Schematic representation of the molecular designs and proof-of-concept models discussed in this work.

Multi-resonance thermally activated delayed fluorescence (MR-TADF) materials based on rigid polycyclic aromatic frameworks show extraordinary sharp emission spectra owing to the inherent structural vibration inhibition and electronic transition characteristics.^1–7^ Due to their outstanding advantage of high color purity, MR-TADF materials have been extensively used in organic light-emitting diodes (OLEDs) with high-definition displays.^8^ To date, boron/nitrogen (B/N)-embedded polycyclic aromatic hydrocarbons (PAHs) have been proven to be the most recognized multiple-resonance (MR) skeletons, and the MR effect manifests in short-range charge-transfer (SRCT) transitions between the atomically separated lowest unoccupied molecular orbital (LUMO) and highest occupied molecular orbital (HOMO) in boron and nitrogen atoms, respectively, which was first reported by the Hatakeyama group called DABNA-1 (Fig. 1).^1^

To improve the luminescence performance and achieve full-color tuning, a series of highly efficient MR-TADF emitters have been designed by introducing and amplifying the influence of peripheral units attached to the *para* site of the B-substituted phenyl ring in the MR-core (*i.e.*TBN-TPA, CzBN3, DtCzB-DPTRZ, *etc.*).^9–11^ The incorporation of peripheral modification units with electron donating groups (EDGs) and electron withdrawing groups (EWGs) significantly narrows the energy gap and improve the emission oscillator strength (*f*), leading to a bathochromic shift in emission and increased radiative rates, without significantly compromising color purity (*i.e.* narrowband emission characteristics were maintained).^12–16^ The incorporation of an asymmetrically fused carbazole with extended π-conjugation introduces an additional *para*-(N–π–N) configuration, which not only further red-shifts the emission but also helps maintain narrowband emission for high color purity, as demonstrated in emitters such as CzCzB and BNTPA.^17,18^ Furthermore, MR-TADF emitters designed by conjugating and fusing multiple B/N skeleton units with *meta*- or *para*-(N–π–N) conjugations have achieved ultra-narrow emission and full-tuning (*i.e.*CzB4, *v*-DABNA, CzB4-*o*Ph, BBCz-R, *etc.*).^19–23^ Significant efforts have been made to modify B/N-skeleton-based MR-TADF emitters for color tuning^20,24,25^ and explore the underlying physical mechanism.

Color tuning through chemical modification of the B/N skeleton can typically be categorized into two main strategies: (i) peripheral modification by substituting bulky donor/acceptor (D/A) groups, which introduce long-range charge-transfer (LRCT) characteristics,^26–31^ and (ii) fusing D/A fragments into the rigid PAH framework to extend the π-conjugation.^32–37^ Since the LUMO and HOMO are offset by one atom in MR skeletons, the wave function of frontier orbitals becomes very sensitive to the site of peripheral modification and skeleton fusion, eventually leading to significant change in photophysical properties (*i.e.*Δ-DABNA-TB).^38^ Therefore, when modifying the B/N skeleton for color tuning purposes, it is crucial to consider the effectiveness of the modification sites. The primary consideration is controlling the lowest emission state energy, *i.e.*, inducing red or blue shifts, which are typically linked to how skeleton modifications influence the π-electronic conjugation.^14,30,39^ Ensuring that the unique narrowband emission characteristics of MR-TADF materials are preserved after the modification is equally important. Achieving this often involves a trade-off between changes in MR characteristics and various factors, such as vibronic coupling, skeleton flexibility, excited-state structural relaxation, *etc.*^40–46^ However, the physical mechanism underlying full-color regulation of MR-TADF emitters through peripheral modification and skeleton fusion remains incompletely understood.

We previously reported wide-range color-tuning MR-TADF frameworks, 7,10-di-*tert*-butyl-5-mesityl-5*H* benzo[5,6][1,4] azaborinin-o[3,2,1-*jk*]carbazole (BIC), composed of 9-methyl-10-mesityl-9,10-dihydro-9-aza-10-boraanthracene (ABA) and 3,6-di-*tert*-butylcarbazole (TCz) (Fig. 1).^47^BIC-mCz and BIC-pCz were obtained by introducing TCz on the BIC core with *meta*- and *para*-(N–π–N) configurations, respectively. mDBIC (*meta*-(N–π–N) conjugation) and pDBIC (*para*-(N–π–N) conjugation) were obtained by conjugatively fusing dual BIC moieties with mirror symmetric and centrosymmetric geometries, respectively. In this study, we utilize the above two sets of peripheral modification and skeleton fusion isomers as proof-of-concept models to comprehensively demonstrate the impact of the modification site on MR-TADF color tuning. Through quantum chemical calculation and time-resolved spectroscopy, we focus on examining how the following factors influence color regulation: (1) conjugative and non-conjugative connection in peripheral modification and skeleton fusion, (2) the detrimental effect of conjugative skeletal expansion on MR properties, (3) the trade-off between SRCT and LRCT characteristics in the lowest emissive state, (4) vibronic coupling during emission transitions, and (5) skeleton flexibility and excited-state structural rearrangement.

## Results and discussion

### Basic photophysics

**Fig. 2:**
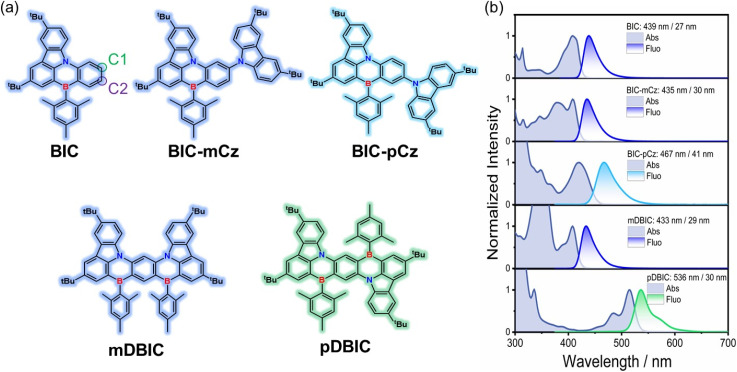
(a) Chemical structures of BIC and its derivatives, with shadow colors approximately corresponding to the fluorescence emission colors of each compound. (b) Normalized stationary absorption and fluorescence spectra of BIC and its derivatives in toluene. The emission wavelength and FWHM are indicated on the fluorescence spectra. Fluorescence spectra were measured under excitation at 360 nm.

Fig. 2 presents the stationary absorption and the emission spectra of BIC and its derivatives. The BIC core displays a narrow absorption band centered at 408 nm, which is attributed to the ^1^SRCT absorption with MR properties. Upon peripheral modification with TCz (an EDG) in a *meta*-(N–π–N) configuration, as seen in BIC-mCz, a new broad absorption band emerges at around 390 nm, corresponding to the ^1^LRCT absorption (see the simulated UV-vis absorption spectra in Fig. S1† and the corresponding electronic excitation analysis). Notably, BIC-mCz retains the sharp ^1^SRCT absorption characteristics of the BIC core. A similar trend is observed in mDBIC, where the fusion of TCz in a *meta*-(N–π–N) configuration within the BIC skeleton leads to analogous spectral changes. In contrast, BIC-pCz and pDBIC, which feature peripheral modification and skeleton fusion of TCz in a *para*-(N–π–N) configuration, respectively, exhibit distinct shifts in the lowest absorption band. BIC-pCz exhibits a broadening of its lowest absorption band, attributed to the introduction of a low-energy ^1^LRCT transition. Meanwhile, pDBIC maintains narrowband characteristics, but its lowest absorption band shifts to approximately 510 nm.

Upon interrogating the emission spectra (Fig. 2b), both BIC-mCz and mDBIC in toluene retain the narrowband properties of the BIC core, with a slight blueshift to 435 and 433 nm, respectively (see Table S1† for detailed photophysical parameters). In contrast, BIC-pCz exhibits a noticeable redshift and broadening emission (*λ*_em_ = 467 nm, PL FWHM = 41 nm or 1806 cm^−1^) compared to the BIC core (*λ*_em_ = 439 nm, PL FWHM = 29 nm or 1482 cm^−1^). This suggests that the MR features in *para*-(N–π–N) substituted BIC-pCz are diminished in both absorption and emission processes. Furthermore, BIC-pCz exhibits significant solvatochromism, with pronounced changes in its emission center and linewidth as a function of solvent polarity (Fig. S2 and Table S1†). In contrast, BIC-mCz, mDBIC, and pDBIC display no more significant solvent dependence in their emission spectra than the MR core, BIC. A redshifted emission with a sharper linewidth (*λ*_em_ = 536 nm, PL FWHM = 30 nm or 1037 cm^−1^) is observed in pDBIC compared to its BIC core (*λ*_em_ = 439 nm, PL FWHM = 29 nm or 1482 cm^−1^). Besides, all modified MR emitters except pDBIC exhibit delayed fluorescence properties in 2 wt%-doped mCP films. The corresponding photophysical parameters are presented in Table S2.† In electroluminescence (EL), the emission spectra exhibit slight broadening and the FWHM of BIC-mCz, BIC-pCz, mDBIC and pDBIC is 42, 48, 42 and 30 nm, respectively (Table S3†).

Based on the above steady-state spectral characteristics, we here preliminarily establish the following trends regarding the site effects on color tuning: (1) the *meta*-(N–π–N) configuration promotes a blueshift, applicable to both peripheral modification and skeleton fusion; (2) the *para*-(N–π–N) configuration promotes a redshift in skeleton fusion, while the emission redshift in peripheral modification is achieved at the expense of narrowband emission properties. To fully understand the color tuning mechanism in MR-TADF emitters, we thus mainly consider the following three aspects: (1) the inheritance and regulation of the original MR characteristics through peripheral modification and skeleton fusion, (2) the influence of flexible EDG introduction at different sites of the MR skeleton on the transition nature of the lowest emission state, and (3) the structural rearrangement in the excited states and its impact on the electron-vibrational coupling and emission linewidth. To understand the importance of these factors, we thus utilize electronic excitation analysis to understand the preservation and modification of MR characteristics, as well as time-resolved spectroscopy to directly characterize the stabilization process of the lowest excited state and the accompanying energy loss.

### Frontier molecular orbitals and transition analysis

The MR effect of the B/N skeleton leads to the formation of non-bonding frontier molecular orbitals (FMOs), which effectively reduce vibronic coupling and vibrational relaxation in the excited-state relaxation. This, in turn, facilitates the realization of highly efficient and narrowband emission. FMO and electron excitation analysis based on density functional theory (DFT) and time-dependent DFT (TD-DFT) provides simple and intuitive insight into the MR effect and the transition characteristics of the lowest excited states when discussing the peripheral modification and skeleton fusion expansion.

**Fig. 3:**
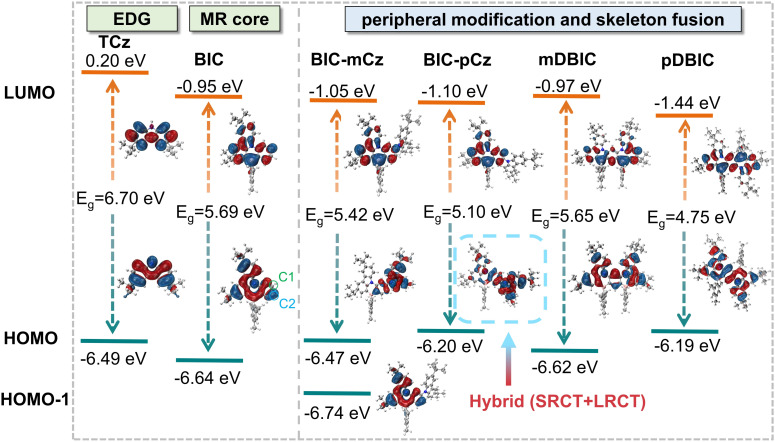
Frontier molecular orbital (FMO) diagrams of BIC and its derivatives. Calculations were performed at the theoretical level of TD-M062X/6-31G(d,p) at their optimized S_0_ geometries.

As shown in Fig. 3, the lowest unoccupied molecular orbital (LUMO) of the MR skeleton core, BIC, exhibits remarkable nonbonding characteristics, with electron density primarily localized on the boron atom and its *ortho* and *para* positions. The highest occupied molecular orbital (HOMO) is distributed on the nitrogen atoms, and exhibits delocalization over the PAH skeleton. The electron–hole analysis of the S_0_ → S_1_ transition, which is predominantly contributed by the HOMO → LUMO transition, also reveals typical SRCT excitation characteristics (see Section S3 in the ESI† for the detailed excitation analysis).

Our previous study demonstrated that when D and A moieties are connected at a void position, where the frontier orbitals from the donor and acceptor are not distributed, a blue-shifted emission is realized compared with the conjugated D–A isomer.^48^ The mechanism is explained as follows: when the conjugative effect is highly suppressed, the originally weaker inductive effect between D and A groups becomes dominant, causing the HOMO energy level to decrease and the LUMO energy level to rise, facilitating a larger energy gap in the D–void–A motif. Herein, BIC-mCz and BIC-pCz were designed by modifying the BIC core at the C1 and C2 positions. Given that TCz acts as an electron donor in these BIC derivatives, we focus on the distributions and energies of HOMOs after chemical modifications. Due to electron density regulation induced by the MR effect of the B/N skeleton, a significant difference in electron density is observed between the C1 (0.12%) and C2 (8.39%) positions in the HOMO of the BIC core (Fig. 3 and S8†), as quantified using the Mulliken population method^49^ with cross terms equally divided by the basis functions in the 6-31(d,p) set. Consequently, the C1 position acts as an ideal void site, whereas the C2 positions act as a conjugative site in constructing TCz-modified derivatives.

When TCz was introduced on the BIC core with the C1 position (*meta*-position) to form BIC-mCz, there are negligible electron density overlaps in the C1 position of the HOMO, thereby forming a D–void–A motif. Although a new HOMO localized on the TCz moieties is introduced in BIC-mCz, excitation analysis indicates that the S_0_ → S_1_ transition in BIC-mCz is predominantly contributed by the HOMO−1 → LUMO transition (Table S5†). The HOMO−1 (−6.74 eV) in BIC-mCz completely inherits the electron distribution characteristics of the HOMO (−6.64 eV) of BIC. As a result, the S_0_ → S_1_ transition in BIC-mCz still exhibits typical SRCT_BIC_ transition characteristics (see the electron–hole analysis in Section S3 in the ESI†) with slightly higher excitation energy (3.67 eV) compared with that of BIC (3.68 eV). These calculation results are consistent with the observed spectral blueshift in the steady-state spectra, which is attributed to the inductive effect in a D–void–A motif when the conjugative effect is suppressed.

In contrast, BIC-pCz is formed by introducing TCz on the BIC core at the C2 position (*para*-position). In this case, the electron density of BIC and TCz can be effectively overlapped in BIC-pCz to form a conjugated D–π–A motif. The strong conjugative effect introduces a new HOMO delocalized over the connected BIC and TCz skeleton. Natural transition orbital (NTO) analysis further indicates that the S_0_ → S_1_ transition (3.43 eV) in BIC-pCz is mainly attributed to hybrid SRCT_BIC_/LRCT_TCz→BIC_ characteristics (Fig. S5†). From the perspective of color tuning, the peripheral modification at the conjugative site (*i.e.* the *para*-(N–π–N) configuration), realizes a redshift in the transition energy.

Furthermore, similar results and detailed theoretical analysis were also reported in other studies.^50,51^*m*-Cz-BNCz and *p*-Cz-BNCz are formed by respectively introducing the TCz into *meta*-/*para*-positions of boron in a blue MR core, BNCz. Due to the change in HOMO and LUMO distributions, *p*-Cz-BNCz exhibits identical HOMO and LUMO distributions and similar energy levels to BNCz, suggesting that *p*-substitution of TCz has negligible influence on the emission properties of BNCz. As a result, *p*-Cz-BNCz exhibits blue-shifted emission compared to BNCz. In contrast, the introduction of TCz at the *meta*-position of boron in BNCz results in the HOMO of *m*-Cz-BNCz being formed by the direct combination of the HOMOs of BNCz and TCz. The HOMO energy level of *m*-Cz-BNCz exhibits a remarkable increase, thereby suggesting a notable red-shift of the emission (*λ*_em_ = 519 nm, green emission).

For isomers mDBIC and pDBIC constructed with skeleton fusion, the fusion sites in a manner are analogous to those in the previous discussion, where mDBIC, with the *meta*-(N–π–N) configuration, can be viewed as an extended D–void–A fusion motif, and pDBIC, with the *para*-(N–π–N) configuration, can be considered an extended D–π–A fusion motif. As expected, the HOMO and LUMO of mDBIC both exhibit clear nonbonding characteristics. Although the orbitals become delocalized over the entire extended skeleton, the calculated HOMO–LUMO energy gap of mDBIC remains essentially unchanged compared to that of the BIC core. In the case of pDBIC, π-electron bonding characteristics are partially observed in both the HOMO and LUMO, particularly at the *para*-(N–π–N) and *para*-(B-π-B) position of the central phenyl. The HOMO–LUMO energy gap of pDBIC is accordingly minimized compared to that of the BIC core.

### Electron distribution regulation by site-specific peripheral modifications

**Fig. 4:**
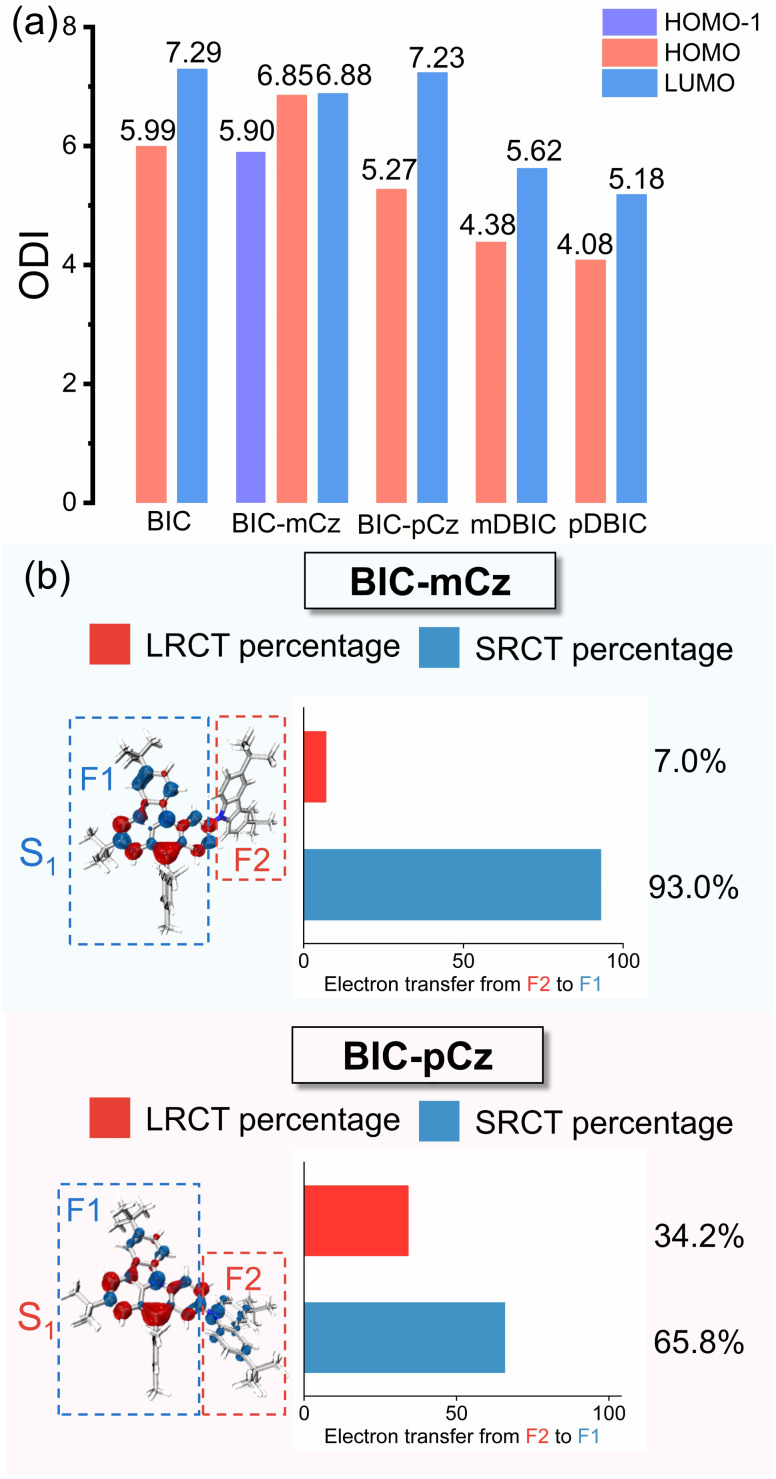
(a) The orbital delocalization index (ODI) analysis of BIC and its derivatives. (b) Hole–electron distributions (hole: blue; electron: red) with inter-fragment charge-transfer (IFCT) analysis of (a) BIC-mCz and (b) BIC-pCz, respectively. BIC-mCz and BIC-pCz are divided into two fragments, *i.e.* F1 (B/N skeleton unit) and F2 (3,6-di-*tert*-butylcarbazole). All calculations at optimized S_0_ geometries.

Skeleton modifications of B/N emitters for color tuning result in changes in the π-electron delocalization at the molecular level. The orbital delocalization index (ODI) is used to quantify the extent of orbital spatial delocalization, and the ODI value of orbital *i* is defined as:
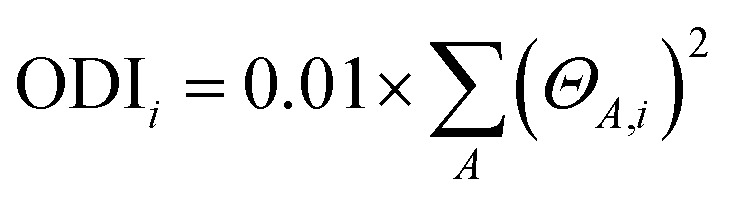
where *Θ*_*A*,*i*_ is the composition of atom *A* in orbital *i*. The ODI value ranges from 0 to 100, with lower values indicating a higher degree of orbital delocalization.^52^ As shown in Fig. 4a, ODI values of HOMO−1 (5.90) and the LUMO (6.88) of BIC-mCz (noted that the S_0_ → S_1_ transition in BIC-mCz is predominantly contributed by the HOMO−1 → LUMO transition) are essentially unchanged compared to that of the HOMO (5.99) and LUMO (7.29) of the BIC core. In contrast, the HOMO of BIC-pCz exhibits an enhanced orbital delocalization with the ODI reduced to 5.27. Meanwhile, the calculation results of nucleus-independent chemical shifts (NICSs), which indicate the aromaticity and delocalization of compounds, also suggest a larger π-electron delocalization in BIC-pCz (lower NICS value at the benzene ring in BIC-pCz) (Fig. S9†). Therefore, in site-specific peripheral modifications, the difference in TCz's orbital involved in the S_0_ → S_1_ transition results in different extents of FMO delocalization.

The interfragment charge-transfer (IFCT) analysis of the S_0_ → S_1_ transition in BIC-mCz and BIC-pCz is used to further quantify the contributions of SRCT and LRCT (see Fig. 4b for fragment partitioning and IFCT results). In BIC-mCz, both the hole and electron distributions are localized on the F1 fragment, exhibiting dominant SRCT_BIC_ characteristics. In contrast, in BIC-pCz, the hole distribution is significantly delocalized across both the F1 and F2 fragments, demonstrating hybrid SRCT_BIC_/LRCT_TCz→BIC_ characteristics. The IFCT analysis reveals that the contribution of LRCT to the S_0_ → S_1_ transition in BIC-pCz increases to 34.2%, compared to only 7.0% in BIC-mCz. Therefore, the D–void–A motif with a *meta*-(N–π–N) configuration is not expected to undergo significant structural rearrangement due to the flexibility of the peripheral EDG, achieving a blue-shifted narrowband emission. The D–π–A motif with a *para*-(N–π–N) configuration, while achieving a red-shifted emission, may experience emission broadening in dielectric environments due to the contribution of LRCT.

### Electron distribution regulation by site-specific skeleton fusion

mDBIC and pDBIC are isomers constructed by fusing dual BIC moieties with *meta*- and *para*-(N–π–N) conjugations, respectively. Compared to the peripheral-modified emitters BIC-mCz and BIC-pCz, both mDBIC and pDBIC exhibit high rigidity in their extended skeletons. As a result, the ODI values of the FMOs for mDBIC and pDBIC indicate that the fusion of the skeleton significantly enhances π-electron delocalization (Fig. 4a). Hole-particle distributions of the S_0_ → S_1_ transition also manifest SRCT characteristics, indicating that TCz has been fully integrated into the molecular skeleton, and the entire molecular structure no longer distinctly separates the electron donor and acceptor segments (Fig. S6 and S7†).

**Fig. 5:**
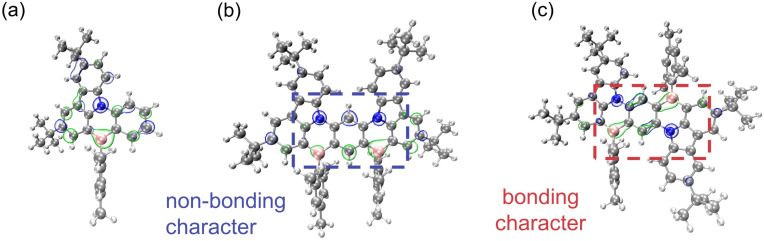
The natural bonding orbital (NBO) analysis of (a) BIC, (b) mDBIC and (c) pDBIC in S_0_ geometries. The blue and green bubbles represent the HOMO and LUMO contribution, respectively.

As mentioned above, the *para*-(N–π–N) configuration presents an extended D–π–A fusion motif, which introduces π-electron bonding characteristics and reduces the MR characteristics. These properties are clearly identified by the natural bonding orbital (NBO) and natural atomic orbital (NAO) analysis (see Fig. 5 and Table S9†). It is found that the π-electrons of mDBIC (*meta*-(N–π–N) conjugation) are localized on the atom, exhibiting non-bonding features. In contrast, the π-electrons of pDBIC (*para*-(N–π–N) conjugation) are localized on the atom and bond, exhibiting more bonding features (especially in the central benzene ring). Therefore, the MR characteristics are maintained or even enhanced in mDBIC, while the MR characteristics in pDBIC are reduced, leading to an emission shoulder at 570 nm (Fig. 2b).

It should be noted that, only pDBIC is centrosymmetric from the perspective of the molecular structure, and structural simulations also show that its central backbone is nearly planar (Fig. S14†). The electron–hole centroid distance of pDBIC is estimated to be about 0.079 Å, much lower than that of other emitters including its mirror symmetric isomer, mDBIC (1.134 Å) (Fig. S15†). Therefore, pDBIC combines the advantages of a highly rigid backbone and a near-zero intrinsic electric dipole moment, which helps suppress emission broadening in dielectric environments, thereby compensating for the potentially increased electron-vibration coupling caused by the decline in MR properties in the *para*-(N–π–N) conjugation.

### Structural flexibility and vibronic coupling on skeleton modification

From the perspective of electron distribution in frontier orbitals, emission redshift modulation *via para*-(N–π–N) modification is inevitably accompanied by a reduction in nonbonding characteristics (*i.e.* MR characteristics). However, the final emission linewidth of the MR-TADF emitter is determined by excited-state structural rearrangements and vibronic coupling of the emission transition, when environmental effects and intermolecular interactions are not considered.

The root-mean-square displacement (RMSD) values provide an appropriate and convenient approach to quantify the overall structural change between the optimized geometries of the S_0_ and S_1_ states (Table 1 and Fig. S16†). For BIC-mCz, despite containing a flexible peripheral EDG, the enhanced MR characteristics in the *meta*-(N–π–N) configuration lead to a suppression of structural rearrangement in the S_1_ state with an RMSD value of 0.16 Å, compared to its BIC core (0.17 Å). In contrast, BIC-pCz exhibits a significantly larger excited-state structural rearrangement (0.42 Å). When examining the dihedral angle between the BIC core and TCz, BIC-mCz exhibits an increase in the dihedral angle from 43° in the optimized S_0_ geometry to 46° in the S_1_ geometry (Fig. S11†), suggesting a decoupling of TCz and the BIC core. This trend supports the proposed inhibition of the conjugation effect by the D–void–A motif. In contrast, BIC-pCz with a D–π–A motif exhibits a reduced dihedral angle as expected from 38.2° to 24.7° (Fig. S12†), indicating a significant enhancement of conjugation in the excited state, which facilitates the ^1^LRCT_TCz→BIC_ state involved in the emission transition.

**Table 1:** Root-Mean-Square Displacement (RMSD) values, reorganization energy, and differences in the intramolecular dihedral angle between the B–N skeleton unit and TCz of BIC derivatives between the S_0_ and S_1_ states

Compound	RMSD [Å]	*λ* [eV]	*θ*_S_1__–*θ*_S_0__/[°]
BIC	0.17	0.21	NA
BIC-mCz	0.16	0.19	3.34
BIC-pCz	0.42	0.33	−13.47
mDBIC	0.24	0.18	2.29
pDBIC	0.08	0.16	0.35

We must note that structural relaxation is inhibited for emitters with fused skeletons, mDBIC and pDBIC, with root-mean-square displacement (RMSD) values of 0.24 and 0.08 Å, respectively (Table 1 and Fig. S16†). The dihedral angle changes between two fused BIC moieties were also negligible (Fig. S13 and S14†). The results about structural rearrangement indicate that extending PAH skeletons enhances the structural rigidity, while the D–void–A fusion motif, although constructed with flexible peripheral modification, also suppresses the excited-state structural rearrangement.

**Fig. 6:**
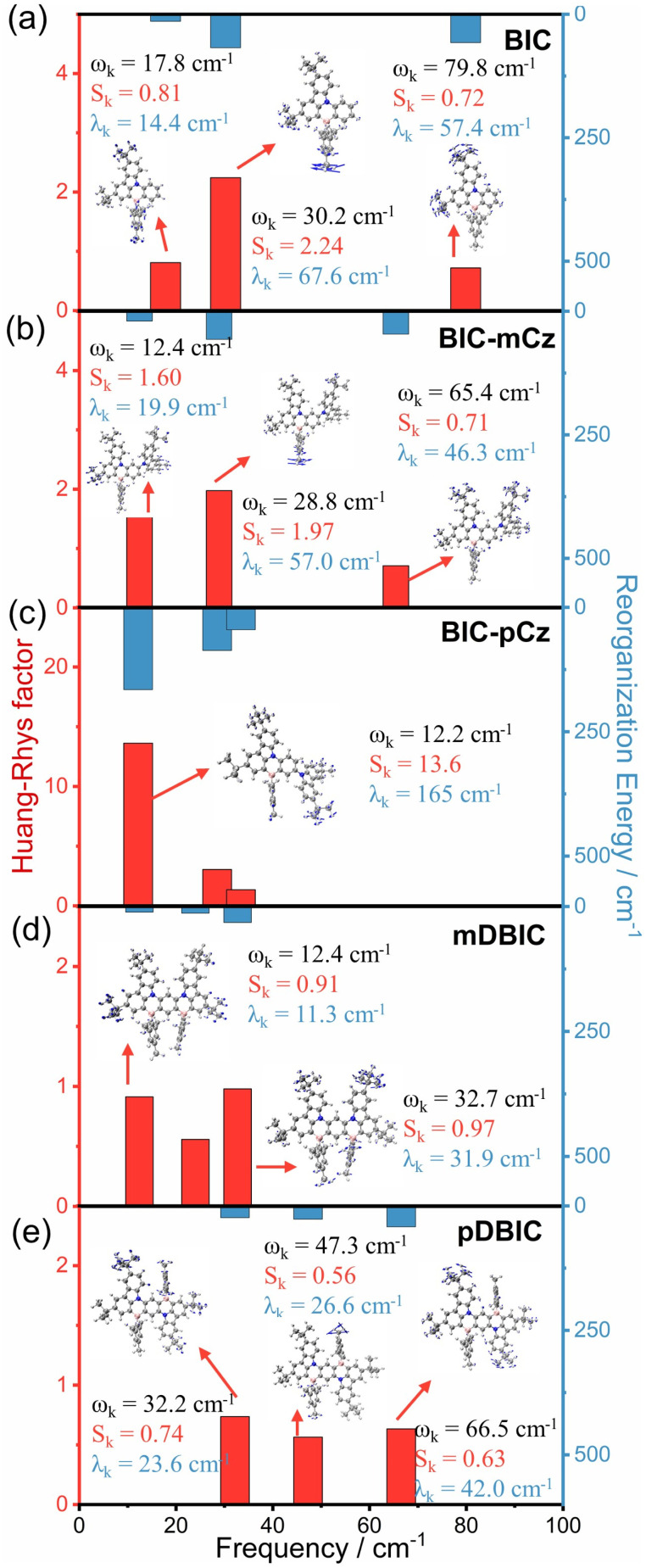
The Huang–Rhys factor (*S*_k_) and reorganization energy contribution (*λ*_k_) of the first three major vibrational modes of (a) BIC, (b) BIC-mCz, (c) BIC-pCz, (d) mDBIC and (e) pDBIC for the S_1_ → S_0_ transition in low frequency regions.

Except for BIC-pCz, the BIC derivatives exhibit low reorganization energies between the S_0_ and S_1_ states, approximately 0.2 eV (Tables 1 and S11†), which facilitate narrow bandwidth emission and lead to a suppression of non-radiative decay processes.^44,53–58^ In this case, the contribution of specific vibrational modes to excited-state structural rearrangements and vibronic coupling during emission transitions could be considered. Vibrational modes with a considerable Huang–Rhys factor (*S*_k_) will be strongly involved in the S_0_ → S_1_ electronic transitions, while the *λ*_k_ is associated with either excited-state structural relaxation or the vibrational motion of the S_1_ state, which broadens the emission linewidth. As shown in Fig. 6, BIC-pCz shows a dominant low-frequency vibrational mode (*ω*_k_ = 12.2 cm^−1^) with an exceptionally large *S*_k_ (13.6) and *λ*_k_ (165 cm^−1^), which is attributed to the dihedral motion between the BIC and *para*-(N–π–N) substituted TCz moieties. In contrast, the major vibrational models in the low-frequency range of the BIC core, *meta*-(N–π–N) substituted BIC-mCz and mDBIC, and *para*-(N–π–N) substituted pDBIC all exhibit small *S*_k_ and *λ*_k_. Additionally, pDBIC shows better suppression of high-frequency modes (2500 to 3500 cm^−1^) compared to its isomer, mDBIC (Fig. S18†). Notably, pDBIC exhibits distinct vibrational modes in the 1200–1500 cm^−1^ range (Fig. S18†), attributed to B/N-embedded acene skeleton stretching vibrations, a feature absent in the other four MR emitters. These modes contribute significantly to its emission profile, likely accounting for the shoulder peak observed in its spectrum.

Therefore, we deduce that for the two emitters regulated in the direction of the long wavelength with *para*-(N–π–N) configurations, peripheral-modification constructed BIC-pCz may exhibit significant excited-state relaxation, while pDBIC, with a centrosymmetric rigid skeleton, may undergo minimal structural relaxation. Furthermore, the SOC constant between the S_1_ and T_1_ state (<S_1_|*Ĥ*_SOC_|T_1_>) in BIC-pCz, due to the mixing LRCT characteristics in the S_1_ state, is significantly enhanced (0.31 cm^−1^) compared with that of other MR emitters (Fig. S20†).

### Excited-state structural rearrangement in skeleton modifications revealed by time-resolved spectroscopy

**Fig. 7:**
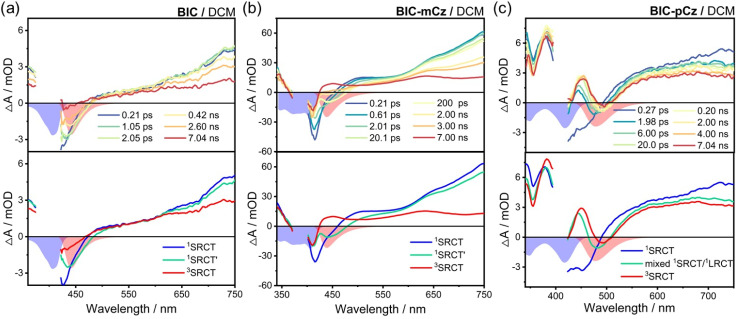
Representative fs-TA spectra of (a) BIC (*λ*_ex_ = 385 nm); (b) BIC-mCz (*λ*_ex_ = 385 nm), and (c) BIC-pCz (*λ*_ex_ = 405 nm) in DCM. Evolution-associated different spectra (EADS) obtained from the global analysis based on a sequential evolution model are shown in the lower panel of the corresponding TA spectra in the 7 ns time window. The fitting time constants are shown in Table S12.† The blue and red shaded areas represent the corresponding steady-state absorption and emission spectra, respectively. SRCT: short-range charge-transfer state, SRCT′: structurally relaxed short-range charge-transfer state, and LRCT: long-range charge-transfer state.

Femtosecond transient absorption (fs-TA) spectroscopy is further employed to gain dynamic insights into the structural relaxation processes of peripheral modification and skeleton fusion,^59,60^ particularly for BIC derivatives with flexible EDG modifications. Fig. 7 shows the representative fs-TA spectra of BIC, BIC-mCz, and BIC-pCz in dichloromethane (DCM). The excitation wavelengths were set to 385 nm for BIC and BIC-mCz, and 405 nm for BIC-pCz, selectively exciting the low-lying ^1^SRCT states and mixed ^1^SRCT/^1^LRCT states, respectively. For the MR core, BIC (Fig. 7a), the initial spectrum at 0.21 ps after photoexcitation shows a positive excited-state absorption (ESA) signal between 500 and 750 nm, which overlaps with the negative ground-state bleach (GSB) below 400 nm and negative stimulated emission (SE) at around 450 nm, as identified by the corresponding steady-state absorption and emission spectra. Following a slight redshift of the SE band by around 7 nm over a few picoseconds, the spectral profile remains stable on a nanosecond scale, until a long-lived species develops. Global spectral analysis (lower panels in Fig. 7) based on a sequential evolution model in the 7 ns time window helps us evaluate the spectral evolution. The rapid stabilization of the SE band indicates a very limited excited-state structural rearrangement in the rigid BIC skeleton.^14,41^ The long-lived species is identified as the ^3^SRCT state based on nanosecond transient absorption (ns-TA) spectroscopy under nitrogen-purged conditions (Fig. S25 and S28†).

We compare the initial spectral evolution of the four BIC derivatives, focusing on the dynamics of structural rearrangement. As expected, the two compounds constructed by skeleton fusion, mDBIC and pDBIC (Fig. S21†), exhibit very minimal SE evolution. This is consistent with the minimal Stokes shift and narrowband emission observed in their steady-state spectra. This can be undoubtedly attributed to excellent skeleton rigidity by the EDG fusion in the BIC parent. For BIC-mCz and BIC-pCz, while both feature a flexible peripheral EDG and share the same BIC core, they exhibit different degrees of SE evolution. As indicated in the electron excitation analysis, peripheral modification in the *meta*-(N–π–N) configuration does not induce significant π-electron delocalization. Consequently, the electron density of the EDG in BIC-mCz is not involved in the emission transition. The emission transition that retained or even enhanced MR characteristics shows minimal sensitivity to solvent polarity. Therefore, the flexible EDG modifications in the *meta*-(N–π–N) configuration do not result in additional excited state structural rearrangement. In contrast, flexible EDG modification in the *para*-(N–π–N) configuration (*i.e.*BIC-pCz) leads to the bonding characteristics at the C–N connection site between the BIC core and the carbazole moiety, and accordingly the MR characteristics are reduced. The π-electron delocalization stabilizes the LRCT state, which replaces the original SRCT state of the BIC core and becomes the lowest emissive state. A sizable electric dipole moment in the S_1_ state results in a remarkable solute–solvent interaction. Therefore, we observe significant SE evolution in central wavelength and intensity, which suggests a substantial excited-state structural rearrangement in the solvent environment, even in weakly polar toluene (Fig. S22†). The proposed excited state relaxation mechanism of BIC derivatives is summarized in Fig. S30.†

## Conclusions

In this study, two sets of peripheral modification and skeleton fusion B/N-skeleton isomers have been investigated as proof-of-concept models to thoroughly demonstrate the site effects in the color-tuning mechanism of MR-TADF emitters. Due to the atomically separated frontier orbital distribution of the core B/N skeleton, the *meta*-(N–π–N) peripheral modification forms a D–void–A motif *via* facilitating the inductive effect and suppressing the conjugative effect, leading to a blue-shift in emission. Meanwhile, LRCT character is prevented from participating in the emission process and thereby maintaining the characteristics of the narrowband emission. This strategy is also applicable to *meta*-(N–π–N) skeleton extension, which can be viewed as an extended D–void–A motif. For the *para*-(N–π–N) configuration, the D–π–A motif is formed *via* a strong conjugative effect and π-electron delocalization, leading to a red-shift in emission. Although the conjugative effect reduces the original non-bonding MR properties, excellent narrowband green emission (pDBIC, 528 nm, 30 nm or 929 cm^−1^ in emission FWHM) is achieved by skeleton fusion of dual BIC in the *para*-(N–π–N) configuration. The rigid centrosymmetric backbone compensates for the potentially increased vibronic coupling caused by the decline in MR properties and further suppresses the emission broadening in dielectric environments induced by the excited-state structural relaxation. Elucidating the site effects in the color-tuning mechanism in B/N-skeleton MR-TADF emitters is poised to make contributions in the following areas: (i) maintaining and enhancing MR characteristics through multiple fusion skeletons or large skeleton extensions;^22^ (ii) fine-tuning of emission colors *via* skeletal modifications while suppressing the emission broadening induced by vibronic coupling or excited-state structural relaxation,^18^ and (iii) addressing the molecular design challenge of achieving ultrapure three primary colors,^37,61^ thus paving the way for future advancements of OLED materials.

## Data availability

The data supporting this article have been included within the article or as part of the ESI.†

## Author contributions

Z. S.: conceptualization, methodology, investigation, data curation, formal analysis, visualization, and writing – original draft; X. L. and S. W.: data curation, formal analysis, and validation; X. W.: resources; Y. L. and Y. W.: data curation and methodology: L. D.: resources and supervision; A. X.: supervision, resources, and funding acquisition; Z. K.: conceptualization, funding acquisition, supervision, and writing – review and editing.

## Conflicts of interest

There are no conflicts to declare.

## Supplementary Material

SC-OLF-D5SC01751C-s001
