# Supplementary material for: The color-tuning mechanism in multi-resonance thermally activated delayed fluorescence emitters: site effects in peripheral modification and skeleton fusion
Source: Chem Sci. 2025 May 20;16(25):11487–97. doi: 10.1039/d5sc01751c (PMC12110348; doi:10.1039/d5sc01751c)
Supplement: SC-016-D5SC01751C-s001 [file SC-016-D5SC01751C-s001.pdf]

Supplementary Information (SI) for Chemical Science.
This journal is © The Royal Society of Chemistry 2025

Supporting Information

Color Tuning Mechanism in Multi-Resonance Thermally Activated Delayed Fluorescence Emitters: Site Effects in Peripheral Modification and Skeleton Fusion

Zicong Situ,[1] Xingqing Li,[1] Shengsheng Wei,[1] Xiang Wang,[2] Yang Li,[1] Yan Wan,[3] Lian Duan,[2,] Andong Xia,[1,*] Zhuoran Kuang[1,*]*

1. State Key Laboratory of Information Photonic and Optical Communications, and School of Science, Beijing University of Posts and Telecommunications (BUPT), Beijing 100876, P. R. China

2. Key Laboratory of Organic Optoelectronics & Molecular Engineering of Ministry of Education, Department of Chemistry, Tsinghua University, Beijing 100084, P. R. China

3. College of Chemistry, Beijing Normal University, Beijing 100875, P. R. China

Corresponding Authors

*Lian Duan	E-mail: duanl@mail.tsinghua.edu.cn
*Andong Xia	E-mail: andongxia@bupt.edu.cn
*Zhuoran Kuang	E-mail: kuang@bupt.edu.cn

Table of Contents

S1. Materials and Methods 3

S2. Supplementary Photophysical Parameters 5

S3. Electronic Excitation Analysis 9

S4. Supplementary Transient Absorption Spectra 24

S5. Supplementary References 32

S1. Materials and Methods

Chemicals. Compounds **BIC**, **BIC-mCz**, **BIC-pCz**, **mDBIC** and **pDBIC** were synthesized as previously reported.[1] All solvents used for spectral measurement are chromatographic pure without further purification.

Stationary Spectral Measurements. Absorption spectra were measured on a spectrophotometer U-3900 (Hitachi, Japan), with an optical density between 0.15 and 0.3 at the peak of the lowest absorption band. Fluorescence spectra were measured on a spectrometer F-4600 (Hitachi, Japan). For fluorescence measurements, the absorbance of the solutions at the band maximum was around 0.1 OD over 1 cm.

Femtosecond Transient Absorption Spectral Measurements. Femtosecond time-resolved transient absorption spectra were measured using a commercial transient absorption spectrometer (Harpia-TA, Light Conversion). Briefly, fundamental pulses are derived from an amplified femtosecond Ti:sapphire laser (Astrella, Coherent). The laser delivers 40 fs pulses at 1kHz and the output is split for white-light continuum generation and optical pumping. The white-light continuum is used as a broadband optical probe from the near-UV to the near-infrared. It is generated by focusing the fundamental laser beam into a 2 mm thick CaF_2 plate, which is oriented and continuously shifted in perpendicular directions. The required pumping pulse is obtained by an optical parametric amplifier (TOPAS-C, Light Conversion). The pump and probe beams were overlapped on a 1 mm thick sample cell and the included polarization angle was set to the magic angle (54.7°) to record the isotropic response. Transient absorption is calculated from consecutive pump-on and pump-poff measurements and averaged over 1000 shots. UV-Vis absorption spectra of the samples are measured before and after every measurement in a spectrophotometer. No significant photodegradation was observed. The femtosecond time-resolved differential absorbance data were analyzed by using R-package TIMP software with the graphical interface Glotaran[2] and CarpetView (Light Conversion). In the global target analysis, the differential absorbances $\Delta A(t, \lambda)$ are decomposed as a superposition of several principal spectral components $\varepsilon_i(\lambda)$ weighed by their concentrations $c_i(t)$: [3]

$$\Delta A(t,\lambda) = \sum_{i=1}^{n} c_i(t)\varepsilon_i(\lambda)$$

Nanosecond Transient Absorption Spectral Measurements. The ns-TA spectra were measured by a commercial spectrometer (Time-Tech Spectra). The generation of the pump beam is the same as that in fs-TA. The probe beam was generated from a supercontinuum laser (LEUKOS-DISCO, French) with the spectral region from 350 to 1800 nm, the repetition rate is 2 kHz, pulse width is 700 ps-1 ns. There is no photodegrading after ns-TA experiments by checking the steady-state absorption spectra.

Quantum Chemical Calculation. The calculations are performed using density generalized function theory (DFT) and time-dependet DFT (TD-DFT) methods with high-nonlocality hybrid functional, which allows for geometric optimization and excitation energy calculations with a 6-

31G(d,p)[4] basis set by fitting parameters that better describe the weak interactions M06-2X.[5] In all the cases, frequency analysis was made after geometry optimization to ensure the convergence to an energy minimum. All simulations were implemented for isolated molecules using the Gaussian 16 software package.[6] The nucleus-independent chemical shifts (NICS) were also calculated by Gaussian 16 software package. Electronic excitation analysis, visualization of electron-hole density, interfragment charge transfer (IFCT) calculations, natural transition orbit (NTO) calculations, natural bond/atomic orbital (NAO/NBO) and orbital delocalization index (ODI) were conducted by the Multiwfn[7] and VMD program.[8] The spin-orbit coupling (SOC) matrix elements between the excited states were calculated by the TD-DFT at the B3LYP-D3/def2-TZVP level without Tamm-Dancoff approximation on the optimized S_0 geometries using ORCA 5.0 program package.[9]

S2. Supplementary Photophysical Parameters

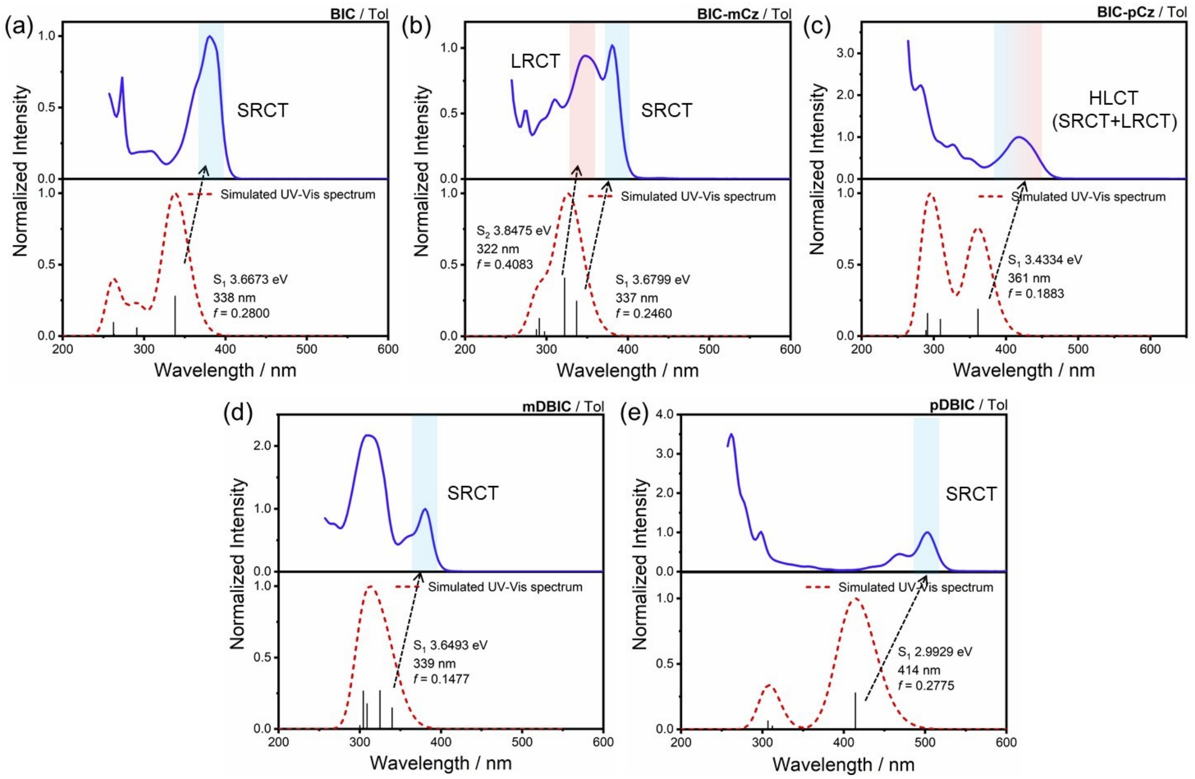

Figure S1. Simulated electronic absorption spectra of (a) **BIC**, (b) **BIC-mCz**, (c) **BIC-pCz**, (d) **mDBIC** and (e) **pDBIC** in Toluene (Tol). The spectrum is simulated based on the TD-DFT vertical excitation energies calculations on the optimized ground-state geometry. The spectral profiles are reconstructed by the GaussView software. The excitation energies of the major transitions and the corresponding oscillator strength (*f*) are depicted in the figure. The calculations were on the theoretical level of M062X/6-31G(d,p) (half-peak width was set as 0.2 eV).

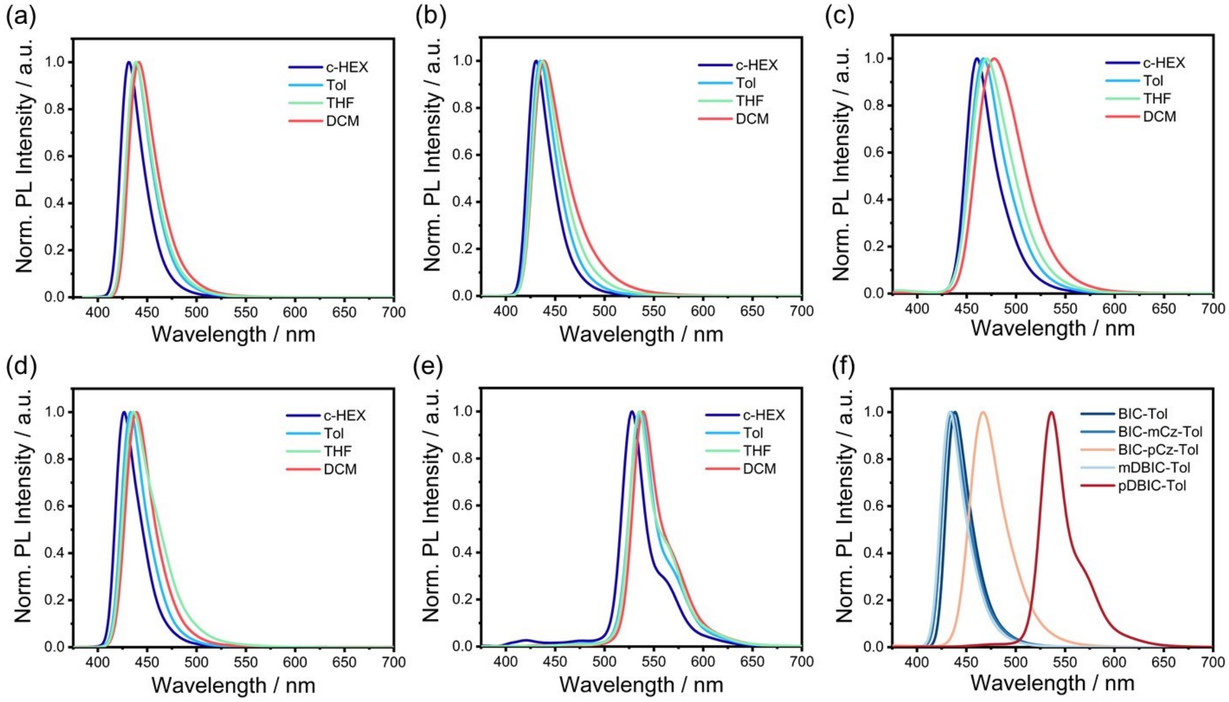

Figure S2. Fluorescent spectra of (a) **BIC**, (b) **BIC-mCz**, (c) **BIC-pCz**, (d) **mDBIC** and (e) **pDBIC** in solvents with different polarities are excited by 360 nm; (f) Normalized spectra in Tol. (cyclohexane (c-HEX), ε = 2.01; toluene (Tol), ε = 2.38; tetrahydrofuran (THF), ε = 7.58; dichloromethane (DCM), ε = 8.93).

Table S1. Photophysical Properties of **BIC** Derivatives.

compound	solvent	λ_{abs} [nm]	λ_{PL} [nm]	PL FWHM [nm]	PL FWHM [cm^{-1}]	Stokes shift [nm]/[cm^{-1}]	solvatochromic shift [nm]/[cm^{-1}]*
**BIC**	c-HEX	408	431	27	1431	23/1308	
	Tol	408	439	29	1482	31/1730	
	THF	408	439	31	1640	31/1730	
	DCM	407	441	33	1665	34/1834	10/526
**BIC-mCz**	c-HEX	408	431	28	1488	23/1308	
	Tol	408	435	30	1509	27/1521	
	THF	407	437	34	1742	30/1687	
	DCM	407	439	37	1931	32/1791	8/423
**BIC-pCz**	c-HEX	420	460	34	1581	40/2070	
	Tol	420	467	41	1806	47/2396	
	THF	419	470	47	2083	51/2590	
	DCM	418	478	54	2312	60/3003	18/819
**mDBIC**	c-HEX	408	427	28	1508	19/1091	
	Tol	408	433	29	1523	25/1415	
	THF	407	436	39	1939	29/1634	
	DCM	407	439	32	1687	32/1791	12/640
**pDBIC**	c-HEX	514	528	27	929	14/516	
	Tol	514	536	30	1037	22/799	
	THF	512	536	31	1069	24/874	
	DCM	513	539	32	1090	26/940	11/387

* The solvatoschromic shift is quantified by comparing the PL centers between the non-polar c-HEX and polar DCM.

Table S2. Photophysical properties of **BIC-mCz**, **BIC-pCz**, **mDBIC** and **pDBIC** in 2 wt%-doped mCP films[a].

Emitter	λ_{em} / nm	Φ_{PF} / %	Φ_{DF} / %	τ_{PF} / ns	τ_{DF} / μs	k_F / 10^7 s^{-1}	k_{IC} / $10^7 s^{-1}$	k_{ISC} / 10^6 s^{-1}	k_{RISC} / 10^3 s^{-1}	ΔE_{ST} / eV
**BIC-mCz**	432	81.2	0.8	5.6	250	14.2	3.2	1.7	4.0	0.29
**BIC-pCz**	471	95.0	1.0	12.5	328	7.7	0.3	0.8	3.1	0.15
**mDBIC**	431	66.7	1.3	6.4	202	10.6	4.9	3.0	5.0	0.31
**pDBIC**	539	85.0	N/A	11.3	N/A	7.5	1.3	N/A	N/A	0.35

[a]all the data have reported in the previous work.[1]

For the fused skeletons isomer, the reorganization energy of **mDBIC** (0.18 eV) is higher than **pDBIC** (0.16 eV). Additionally, **pDBIC** shows better suppression of high-frequency modes (2500 to 3500 cm^{-1}) to its isomer, **mDBIC** (**Figure S18**). Thus, the k_{IC} of **mDBIC** in in 2wt%-doped mCP film is faster than **pDBIC** lead to the PLQY of **pDBIC** (85.0%) is higher than **mDBIC** (66.7%). For the ΔE_{ST} parameter, **mDBIC** (0.31 eV) and **pDBIC** (0.35 eV) have close value due to they are isomers. Besides, **mDBIC** shows delayed fluorescence properties in in 2wt%-doped mCP film while **pDBIC** do not exhibits delayed fluorescence properties. Therefore, it is hard to discuss the k_{RISC} between **mDBIC** and **pDBIC**.

Table S3. EL properties of the OLED devices based on these emitters.[a]

Emitter	λ_{EL} [nm]	EQE_{max} [%]	FWHM [nm]	CIE [x,y]
**BIC-mCz**	432	19.4	42	(0.16, 0.05)
**BIC-pCz**	466	39.8	48	(0.14, 0.16)
**mDBIC**	431	13.5	42	(0.16, 0.05)
**pDBIC**	535	31.0	30	(0.33, 0.64)

[a]all the data have reported in the previous work.[1]

S3. Electronic Excitation Analysis

Table S4. TD-DFT Calculated Electronic Excitation Properties of the **BIC**.

**Electronic Transition**	**Excitation Energy/ eV**	**Oscillator Strength**	**Transitions**
S_0 geometry			
$S_0 \rightarrow S_1$	3.6673	0.2800	H → L (95.2%)
$S_0 \rightarrow S_2$	4.1727	0.0044	H-1 → L (51.5%)<br>H-2 → L (38.6%)
$S_0 \rightarrow S_3$	4.2608	0.0578	H-2 → L (50.1%)<br>H-1 → L (34.2%)
$S_0 \rightarrow S_4$	4.7155	0.0155	H-3 → L (57.2%)<br>H-4 → L (21.3%)
$S_0 \rightarrow S_5$	4.7267	0.0955	H → L+1 (63.3%)<br>H-5 → L (8.8%)
$S_0 \rightarrow T_1$	2.9712	0.0000	H → L (90.5%)
$S_0 \rightarrow T_2$	3.2950	0.0000	H-1 → L (62.5%)<br>H-1 → L+1 (9.7%)
$S_0 \rightarrow T_3$	3.8102	0.0000	H-4 → L (48.9%)<br>H → L+2 (19.6%)
$S_0 \rightarrow T_4$	3.9832	0.0000	H-2 → L (46.5%)<br>H-2 → L+4 (19.0%)
$S_0 \rightarrow T_5$	4.0283	0.0000	H → L+1 (53.3%)<br>H-5 → L (8.2%)

Table S5. TD-DFT Calculated Electronic Excitation Properties of the **BIC-mCz**.

**Electronic Transition**	**Excitation Energy/ eV**	**Oscillator Strength**	**Transitions**
S_0 geometry			
$S_0 \rightarrow S_1$	3.6799	0.2460	H-1 → L (93.4%)
$S_0 \rightarrow S_2$	3.8475	0.4083	H → L (80.7%)<br>H → L+2 (6.2%)
$S_0 \rightarrow S_3$	4.1667	0.0321	H-4 → L (47.2%)<br>H-3 → L (42.0%)
$S_0 \rightarrow S_4$	4.2581	0.1246	H-3 → L (43.6%)<br>H-4 → L (41.8%)
$S_0 \rightarrow S_5$	4.3111	0.0466	H → L+1 (88.2%)<br>H-2 → L+5 (7.2%)
$S_0 \rightarrow T_1$	2.9947	0.0000	H-1 → L (87.2%)
$S_0 \rightarrow T_2$	3.2953	0.0000	H-3 → L (59.1%)<br>H-3 → L+2 (12.3%)
$S_0 \rightarrow T_3$	3.3934	0.0000	H → L (55.2%)<br>H-7 → L (11.9%)
$S_0 \rightarrow T_4$	3.6342	0.0000	H → L+1 (89.1%)
$S_0 \rightarrow T_5$	3.6921	0.0000	H-2 → L+1 (71.3%)<br>H → L+5 (11.1%)

Table S6. TD-DFT Calculated Electronic Excitation Properties of the **BIC-pCz**.

**Electronic Transition**	**Excitation Energy/ eV**	**Oscillator Strength**	**Transitions**
S_0 geometry			
$S_0 \rightarrow S_1$	3.4334	0.1883	H → L (76.8%)<br>H-2 → L (14.4%)
$S_0 \rightarrow S_2$	4.0124	0.1176	H-2 → L (53.1%)<br>H-1 → L (10.1%)
$S_0 \rightarrow S_3$	4.1180	0.0023	H-4 → L (56.4%)<br>H-3 → L (33.6%)
$S_0 \rightarrow S_4$	4.2578	0.1577	H → L+2 (67.1%)<br>H-3 → L (7.6%)
$S_0 \rightarrow S_5$	4.2886	0.0393	H-3 → L (39.1%)<br>H-4 → L (27.8%)
$S_0 \rightarrow T_1$	2.8229	0.0000	H → L (52.8%)<br>H-2 → L (31.2%)
$S_0 \rightarrow T_2$	3.2765	0.0000	H-3 → L (55.1%)<br>H-4 → L (11.2%)
$S_0 \rightarrow T_3$	3.5835	0.0000	H → L+2 (53.4%)<br>H-2 → L+2 (8.3%)
$S_0 \rightarrow T_4$	3.6155	0.0000	H → L+2 (22.8%)<br>H-7 → L (22.0%)
$S_0 \rightarrow T_5$	3.6961	0.0000	H-1 → L+2 (58.9%)<br>H-2 → L+2 (11.0%)

Table S7. TD-DFT Calculated Electronic Excitation Properties of the **mDBIC**.

**Electronic Transition**	**Excitation Energy/ eV**	**Oscillator Strength**	**Transitions**
S_0 geometry			
$S_0 \rightarrow S_1$	3.6493	0.1477	H → L+1 (64.4%)<br>H-1 → L (27.3%)
$S_0 \rightarrow S_2$	3.8161	0.2678	H → L (75.4%)<br>H-1 → L+1 (9.8%)
$S_0 \rightarrow S_3$	4.0125	0.1766	H-1 → L+1 (55.5%)<br>H-2 → L (10.3%)
$S_0 \rightarrow S_4$	4.0745	0.2651	H-1 → L (22.9%)<br>H-2 → L+1 (12.4%)
$S_0 \rightarrow S_5$	4.1311	0.0244	H-2 → L (28.1%)<br>H-5 → L+1 (14.0%)
$S_0 \rightarrow T_1$	3.1059	0.0000	H → L+1 (50.9%)<br>H-1 → L (34.8%)
$S_0 \rightarrow T_2$	3.1846	0.0000	H → L (40.7%)<br>H-1 → L+1 (39.9%)
$S_0 \rightarrow T_3$	3.2933	0.0000	H-2 → L (24.1%)<br>H-2 → L+1 (12.7%)
$S_0 \rightarrow T_4$	3.3272	0.0000	H-4 → L+1 (20.2%)<br>H-3 → L+1 (12.7%)
$S_0 \rightarrow T_5$	3.4235	0.0000	H-1 → L+1 (23.4%)<br>H → L (23.2%)

Table S8. TD-DFT Calculated Electronic Excitation Properties of the **pDBIC**.

**Electronic Transition**	**Excitation Energy/ eV**	**Oscillator Strength**	**Transitions**
S_0 geometry			
$S_0 \rightarrow S_1$	2.9929	0.2775	H → L (95.1%)
$S_0 \rightarrow S_2$	3.6618	0.0002	H → L+1 (45.9%)<br>H-1 → L (43.2%)
$S_0 \rightarrow S_3$	3.9717	0.0258	H-2 → L (51.5%)<br>H-5 → L (14.6%)
$S_0 \rightarrow S_4$	4.0105	0.0046	H-4 → L (50.5%)<br>H-3 → L (29.2%)
$S_0 \rightarrow S_5$	4.0418	0.0650	H-5 → L (63.3%)<br>H-2 → L (6.4%)
$S_0 \rightarrow T_1$	2.3184	0.0000	H → L (88.8%)
$S_0 \rightarrow T_2$	3.0029	0.0000	H → L+1 (40.4%)<br>H-1 → L (38.6%)
$S_0 \rightarrow T_3$	3.2186	0.0000	H-2 → L (36.4%)<br>H-2 → L+1 (15.1%)
$S_0 \rightarrow T_4$	3.2704	0.0000	H-3 → L (36.8%)<br>H-3 → L+1 (24.3%)
$S_0 \rightarrow T_5$	3.4812	0.0000	H-8 → L (46.1%)<br>H → L+3 (19.6%)

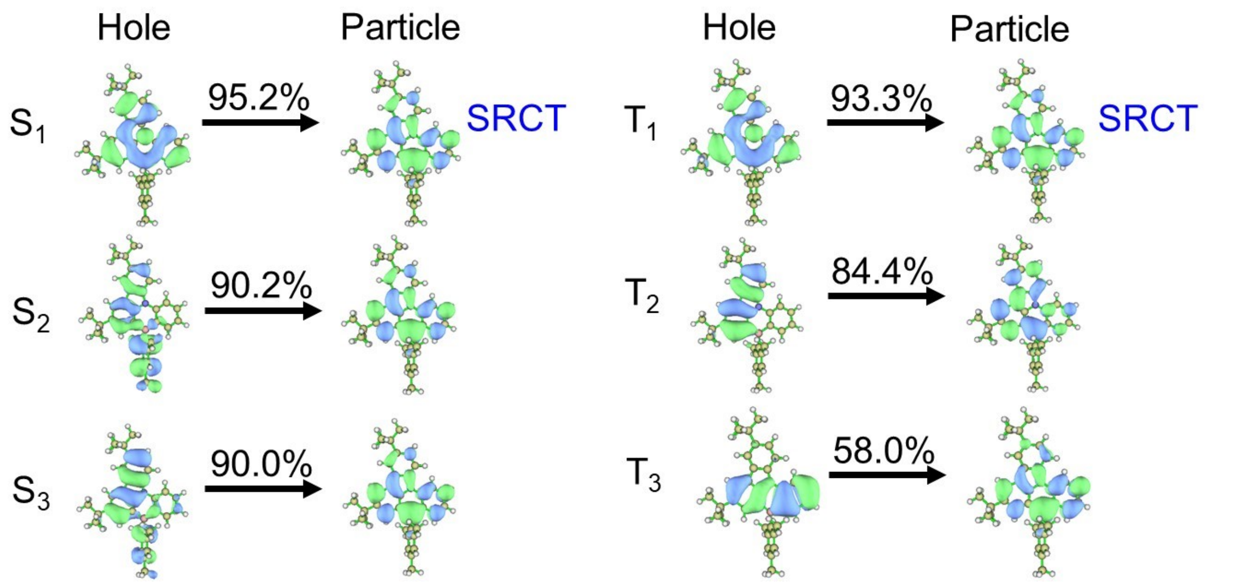

Figure S3. Natural transition orbital (NTO) analysis of **BIC** at the optimized S_0 geometries.

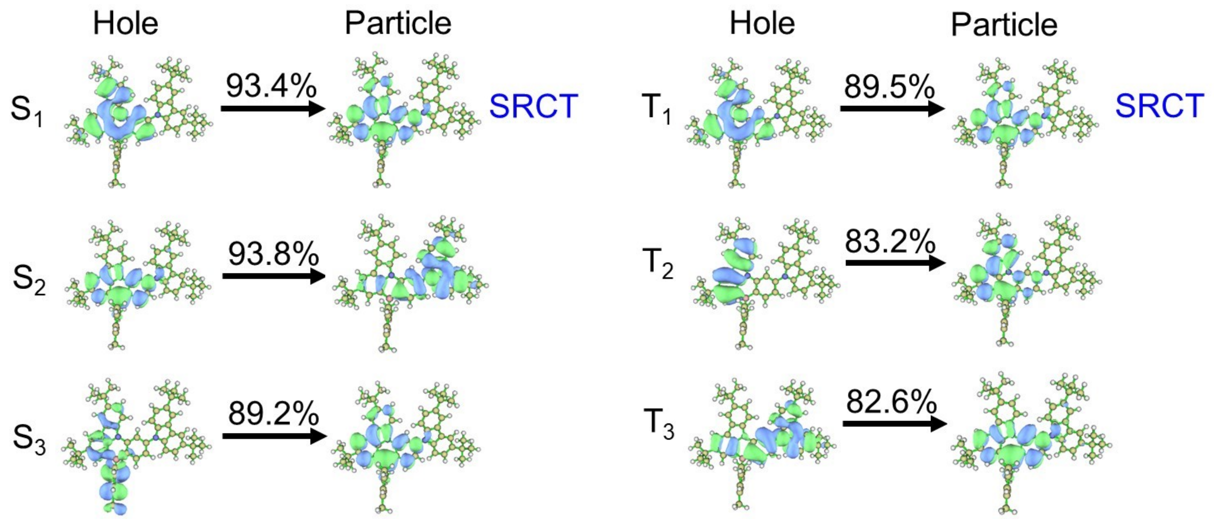

Figure S4. NTO analysis of **BIC-mCz** at the optimized S_0 geometries.

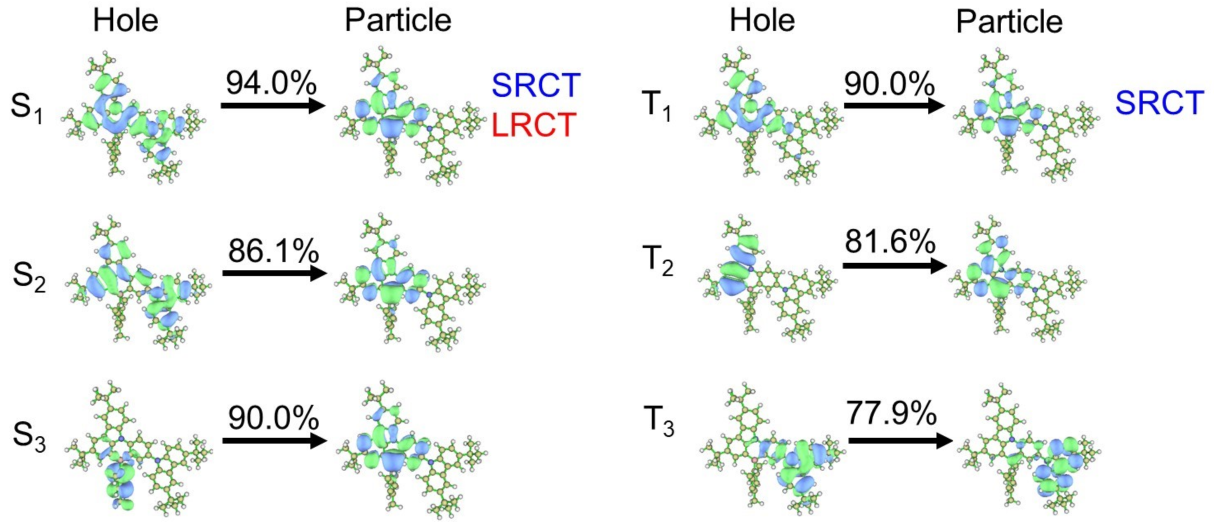

Figure S5. NTO analysis of **BIC-pCz** at the optimized S_0 geometries.

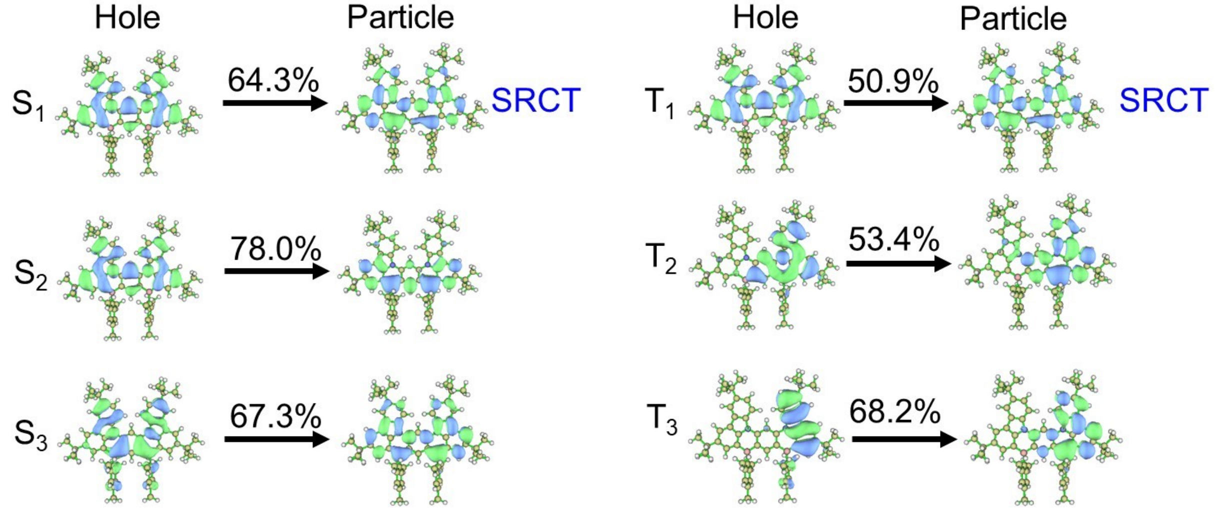

Figure S6. NTO analysis of **mDBIC** at the optimized S_0 geometries.

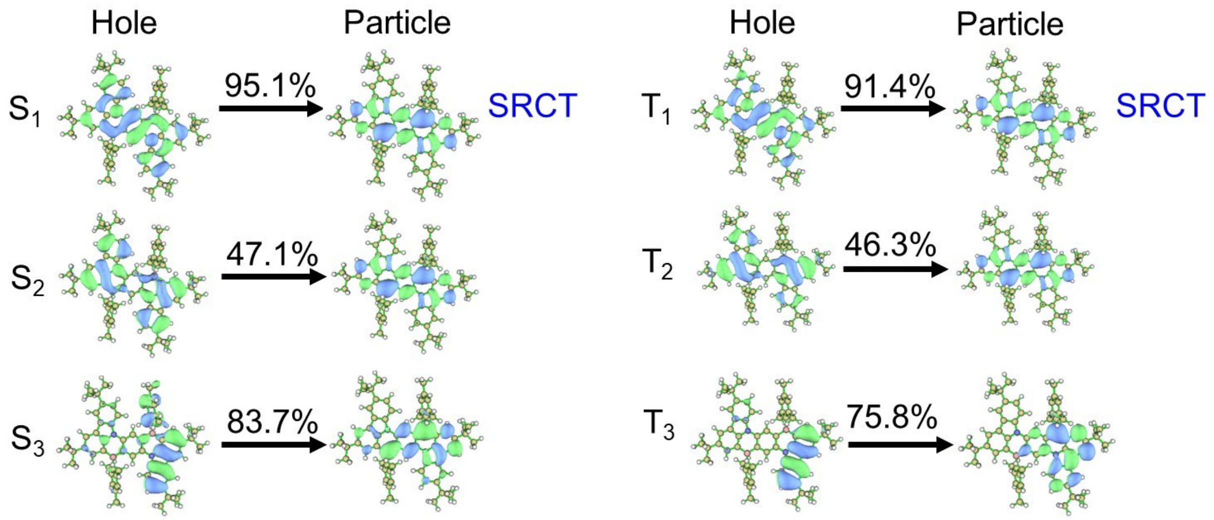

Figure S7. NTO analysis of **pDBIC** at the optimized S_0 geometries.

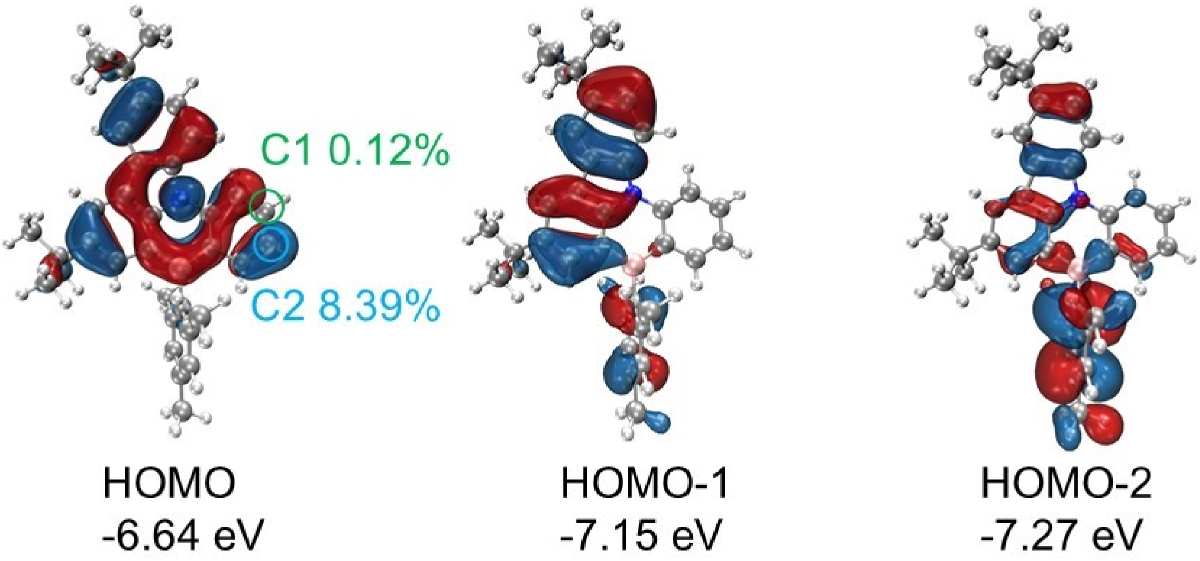

Figure S8. The calculated HOMO, HOMO-1 and HOMO-2 distribution of **BIC** segment. The distribution of C1 and C2 in HOMO was also provided.

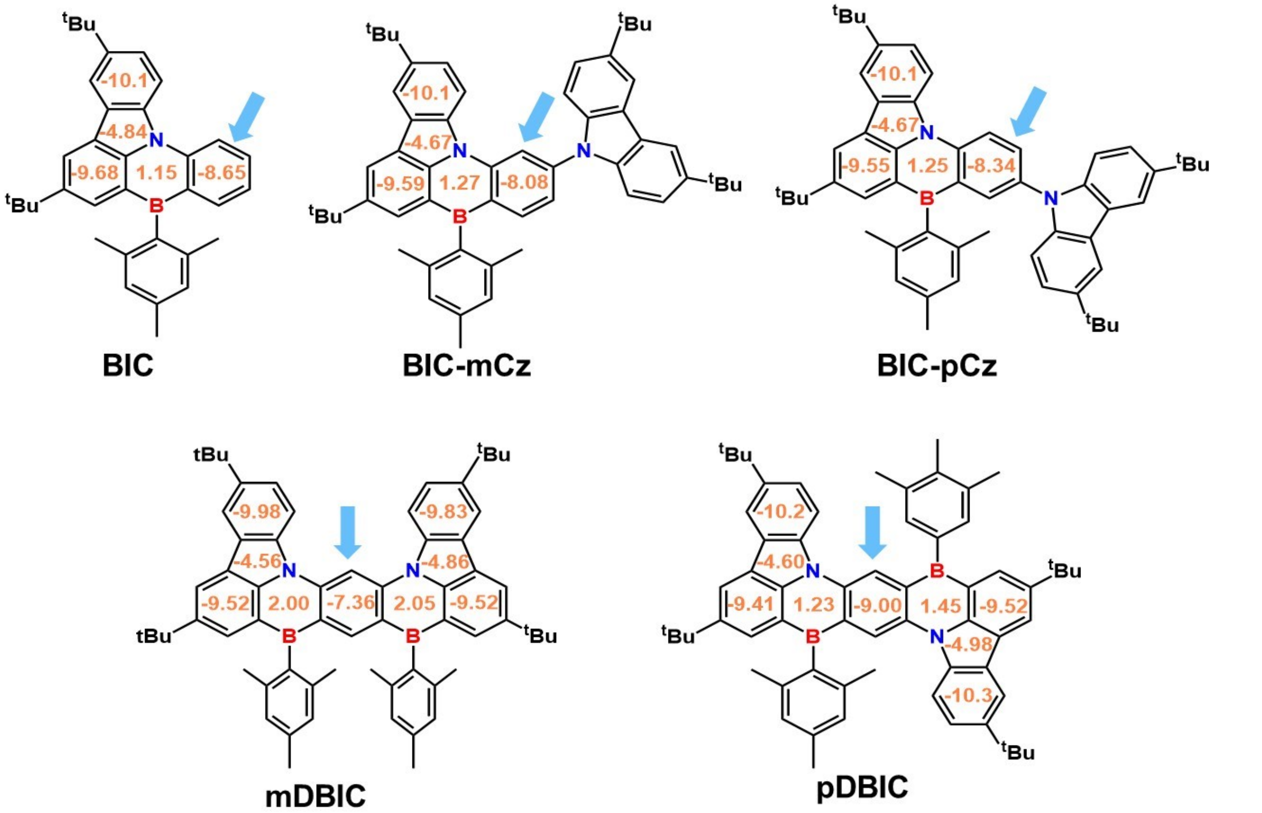

Figure S9. The calculated nucleus-independent chemical shifts (NICS) of **BIC** derivatives. The smaller the value, the stronger the aromaticity and delocalization.

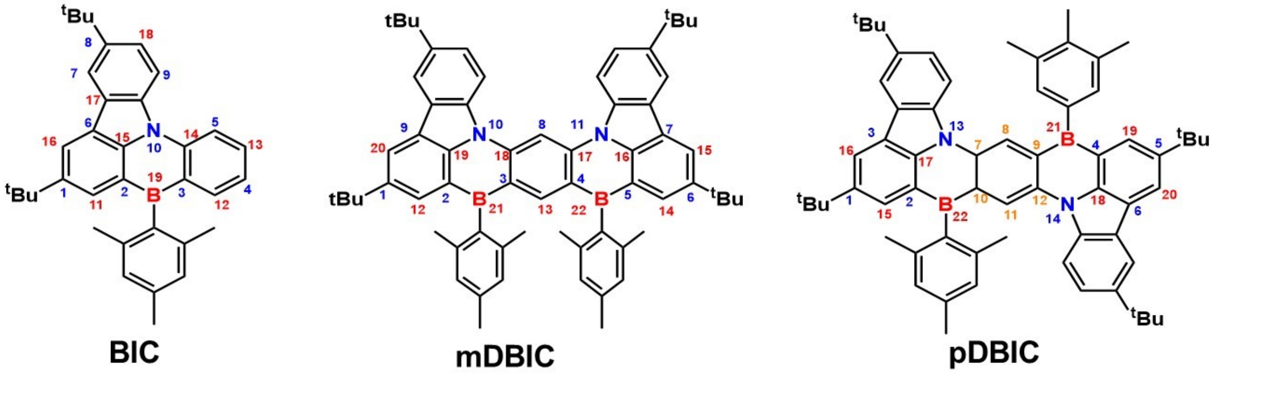

Figure S10. The chemical structure atomic labeling in **Table S9**.

Table S9. Contribution of Natural Atomic Orbitals (NAO) in **BIC**, **mDBIC** and **pDBIC**.

**BIC**		**mDBIC**		**pDBIC**	
lables	contribution / %	lables	contribution / %	lables	contribution / %
		HOMO			
1-C	9.16	1-C	6.16	1-C	3.75
2-C	4.84	2-C	4.43	2-C	3.12
3-C	4.14	3-C	0.23	3-C	2.46
4-C	8.39	4-C	0.51	4-C	2.77
5-C	7.34	5-C	4.26	5-C	3.98
6-C	6.38	6-C	5.39	6-C	3.07
7-C	5.04	7-C	2.64	7-C	4.95
8-C	7.36	8-C	14.5	8-C	6.86
9-C	8.20	9-C	3.33	9-C	1.49
10-N	18.7	10-N	9.51	10-C	1.45
		11-N	10.9	11-C	7.06
				12-C	4.94
				13-N	10.8
				14-N	11.2
		LUMO			
11-C	13.2	12-C	3.62	7-C	1.92
12-C	5.77	13-C	12.9	8-C	6.12
13-C	8.17	14-C	8.59	9-C	5.97
14-C	4.78	15-C	9.47	10-C	6.05
15-C	0.95	16-C	1.71	11-C	5.82
16-C	12.6	17-C	0.07	12-C	2.01
17-C	2.56	18-C	1.36	15-C	4.60
18-C	2.78	19-C	1.69	16-C	5.23
19-B	27.9	20-C	4.77	17-C	1.22
		21-B	11.2	18-C	1.02
		22-B	21.8	19-C	5.34
				20-C	5.07
				21-B	18.0
				22-B	17.0

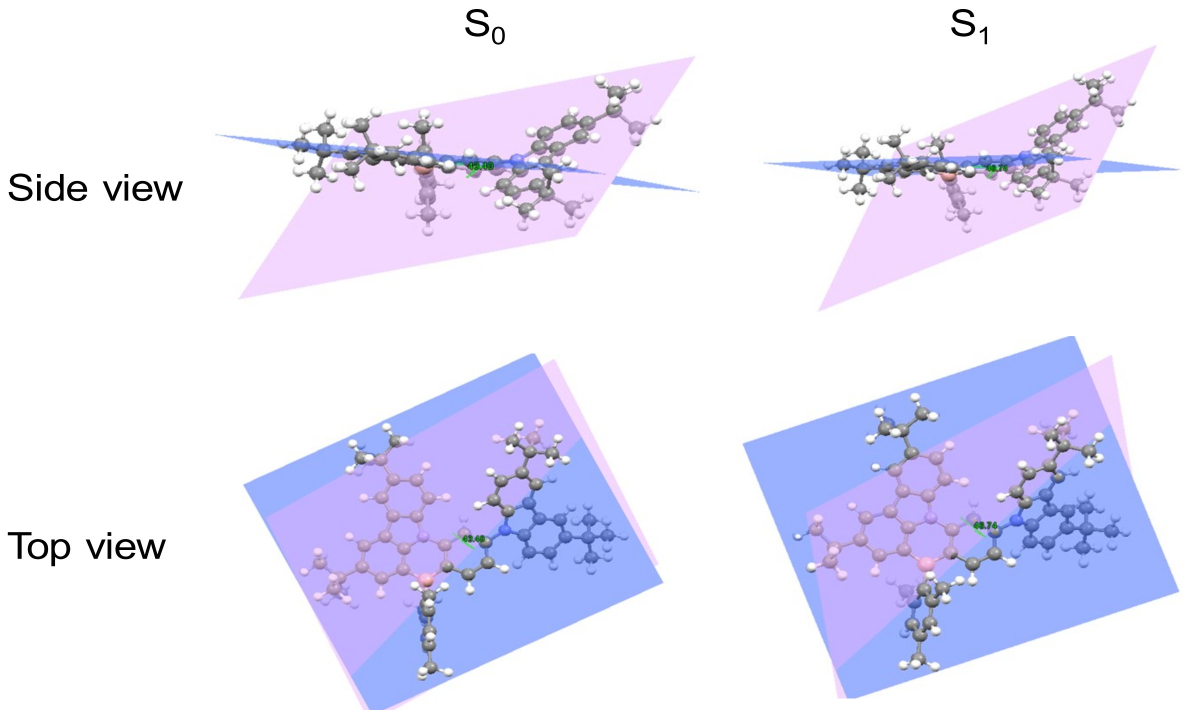

Figure S11. The intramolecular dihedral angles of B-N skeleton unit and carbazole unit in the S_0 and S_1 of **BIC-mCz**.

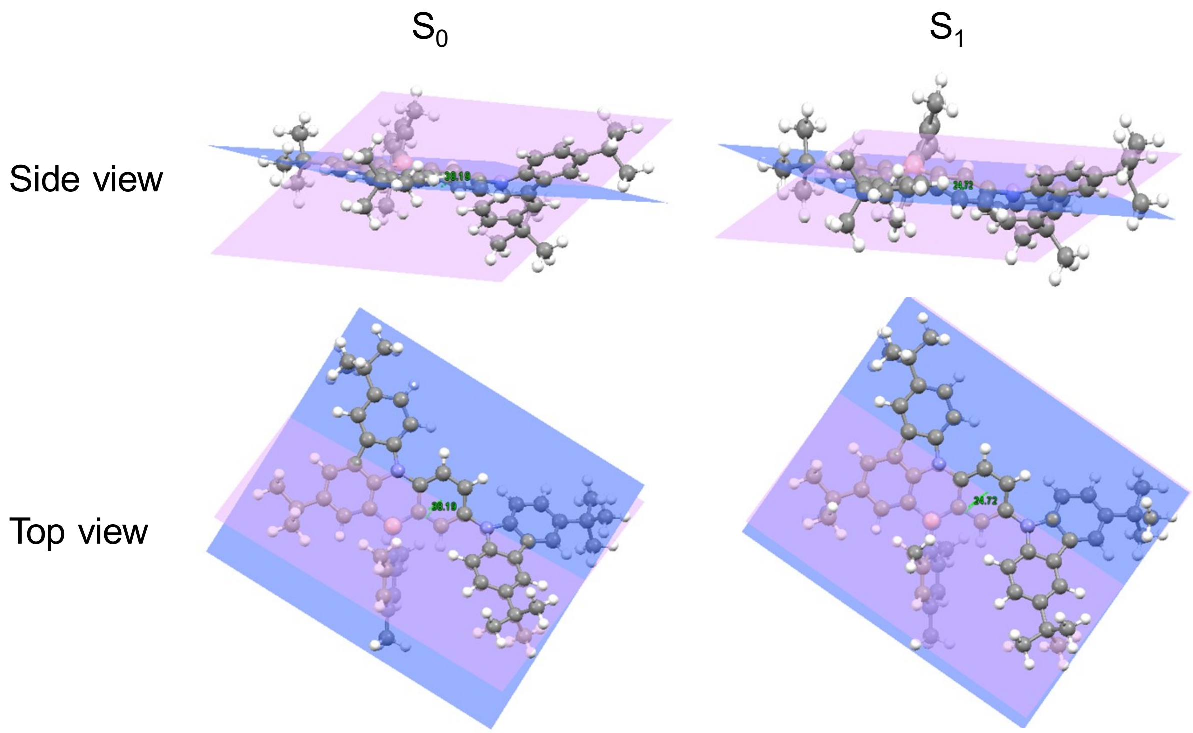

Figure S12. The intramolecular dihedral angles of B-N skeleton unit and carbazole unit in the S_0 and S_1 of **BIC-pCz**.

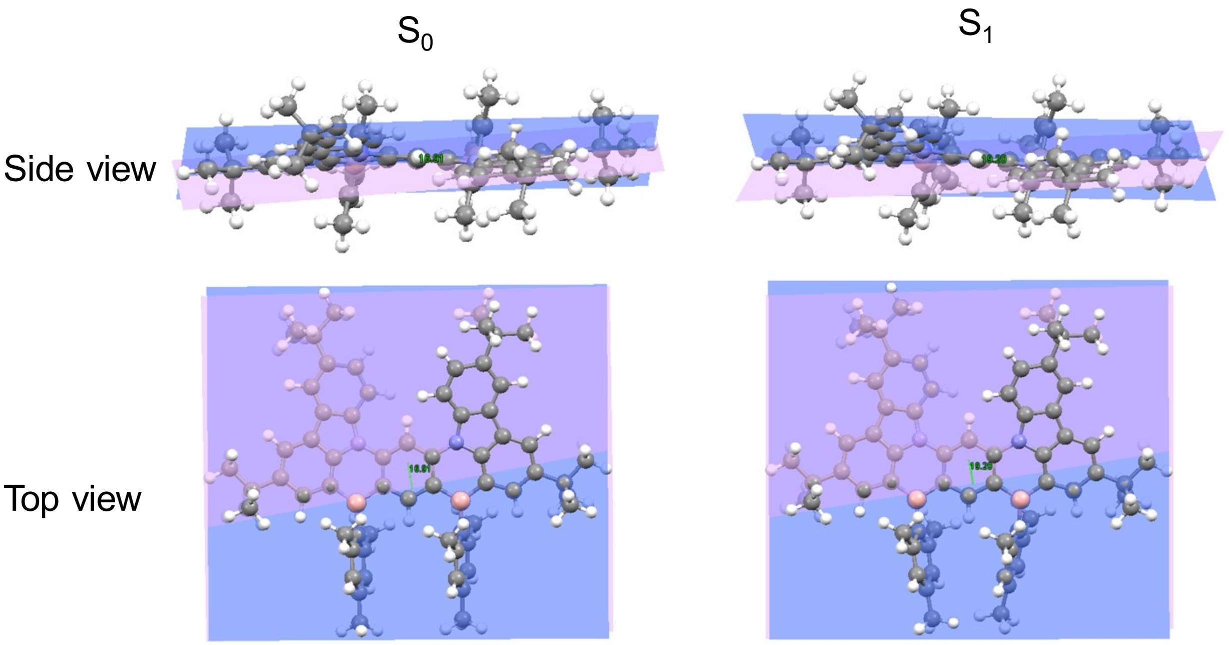

Figure S13. The intramolecular dihedral angles of B-N skeleton unit and carbazole unit in the S_0 and S_1 of **mDBIC**.

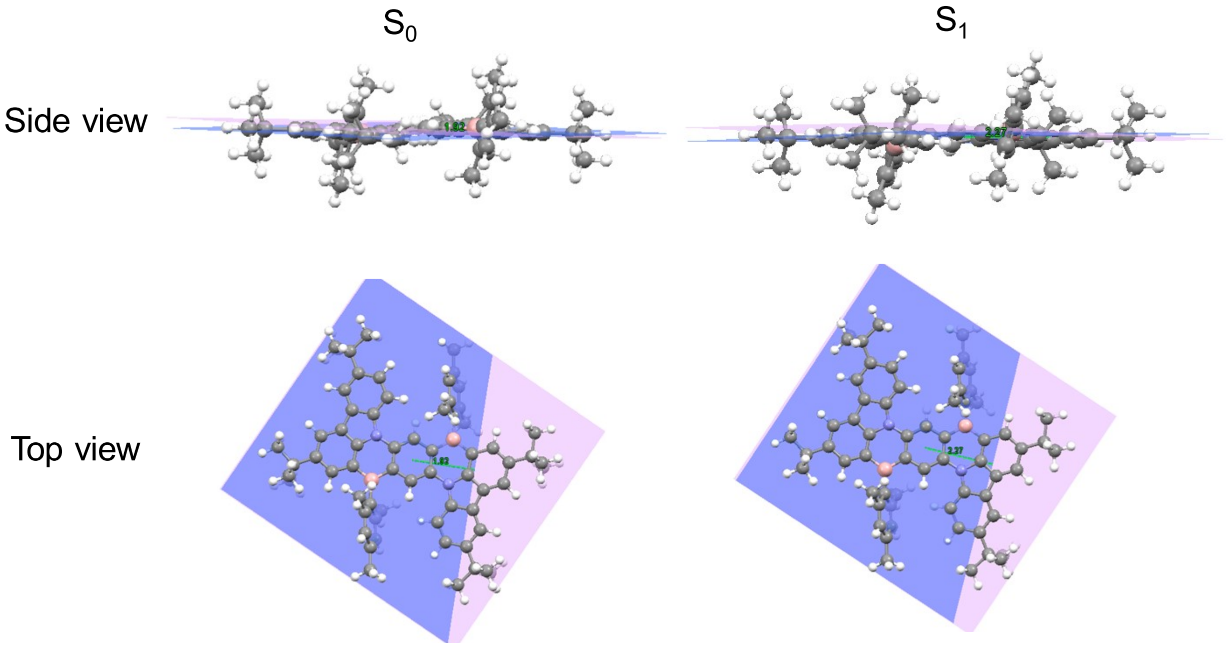

Figure S14. The intramolecular dihedral angles of B-N skeleton unit and carbazole unit in the S_0 and S_1 of **pDBIC**.

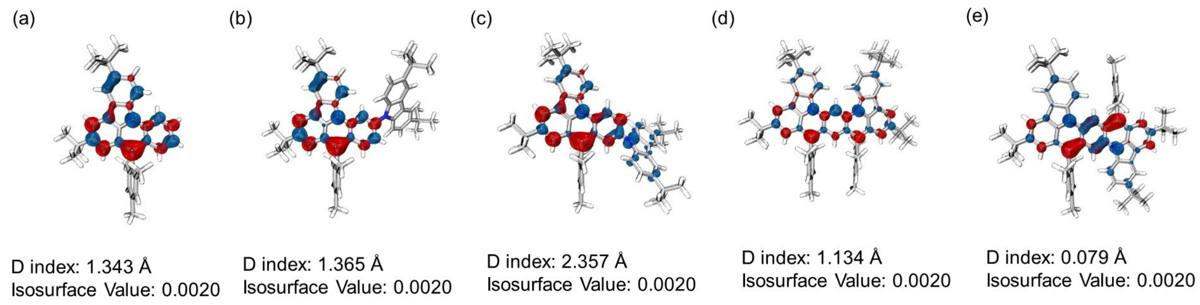

Figure S15. Electron-hole analysis of (a) **BIC**, (b) **BIC-mCz**, (c) **BIC-pCz**, (d) **mDBIC** and (e) **pDBIC** in S_1 state at the optimized S_0 geometries.. The blue and green color represent the hole and electron, respectively.

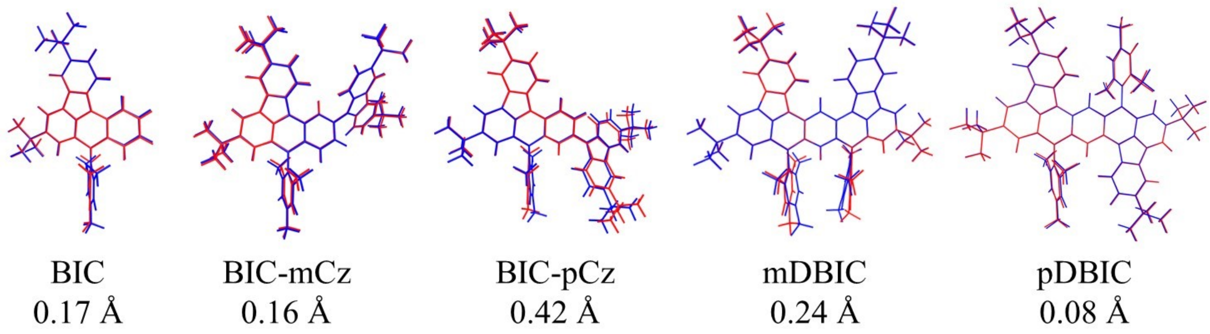

Figure S16. Root-mean-square displacement (RMSD) values of target molecules between S_0 and S_1 state.

Table S10 The Intramolecular Dihedral Angles of B-N Skeleton Unit and **TCz** Unit in the S_0 and S_1 of **BIC-mCz**, **BIC-pCz**, **mDBIC** and **pDBIC**.

**Dihedral angle/°**	**BIC-mCz**	**BIC-pCz**	**mDBIC**	**pDBIC**
θ_{S0}	43.40	38.19	16.91	1.92
θ_{S1}	46.74	24.72	19.20	2.27
$\mid \theta_{S1}-\theta_{S0} \mid$	3.34	13.47	2.29	0.35

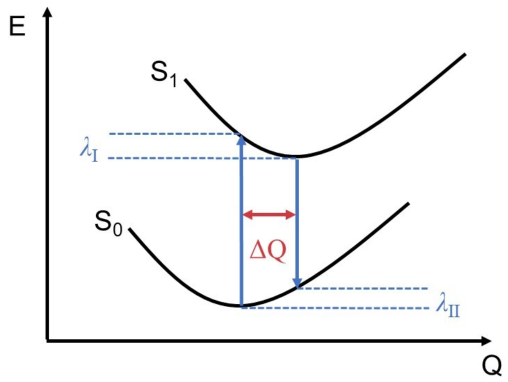

Figure S17. The sketch of the potential energy surfaces for the S_1 and S_0 states, illustrates the normal-mode displacement (ΔQ) and the total relaxation energy λ ($\lambda = \lambda_I + \lambda_{II}$).

Table S11 Reorganization energy of **BIC, BIC-mCz, BIC-pCz, mDBIC** and **pDBIC**.

	λ_I / eV	λ_{II} / eV	$\lambda = \lambda_I + \lambda_{II}$ / eV
**BIC**	0.10	0.11	0.21
**BIC-mCz**	0.09	0.10	0.19
**BIC-pCz**	0.16	0.17	0.33
**mDBIC**	0.10	0.08	0.18
**pDBIC**	0.08	0.08	0.16

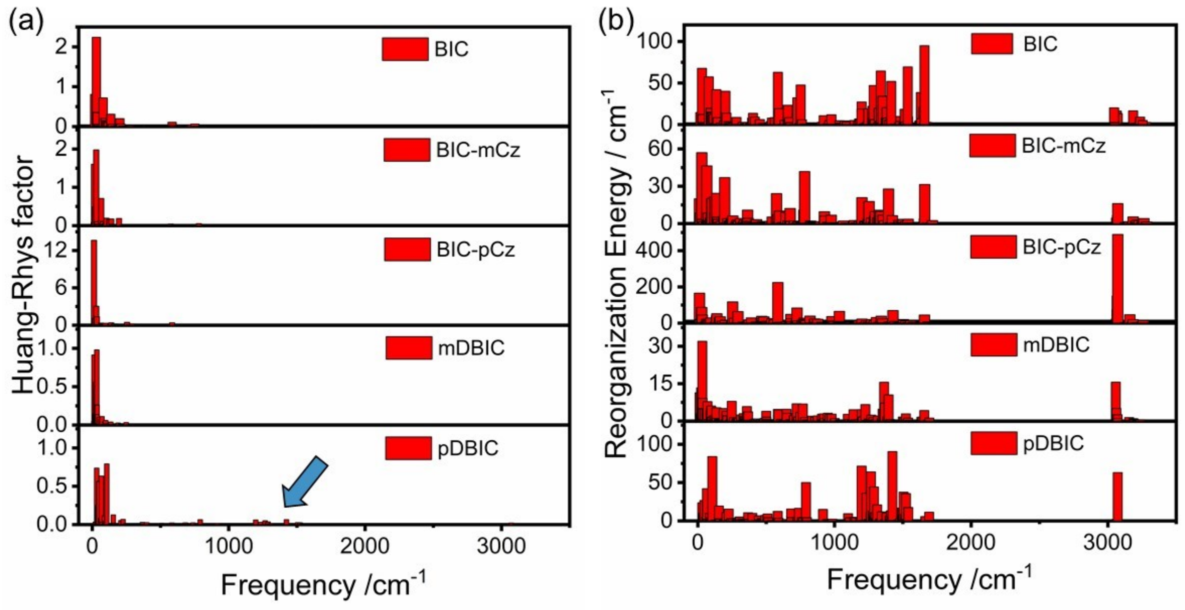

Figure S18. (a) The Huang–Rhys factor (S_k) and (b) reorganization energy contribution (λ_k) of each vibrational mode of **BIC** derivatives for the $S_1 \rightarrow S_0$ transition.

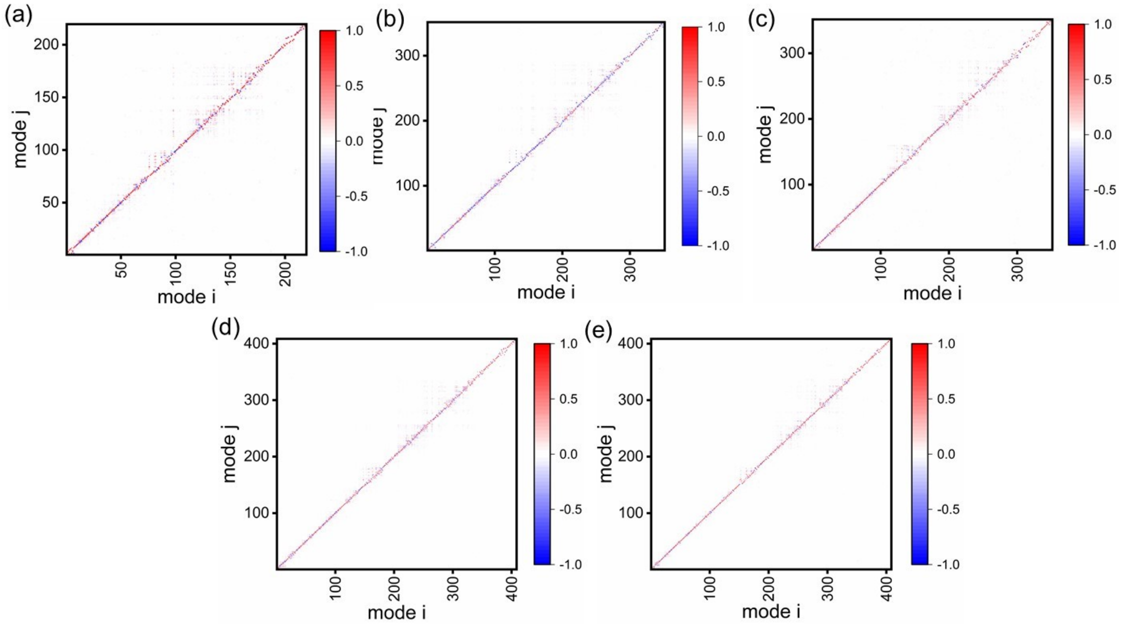

Figure S19. Duschinsky matrix of the 219, 351, 351, 408 and 408 vibrational modes of **BIC**, **BIC-mCz**, **BIC-pCz**, **mDBIC** and **pDBIC**, respectively.

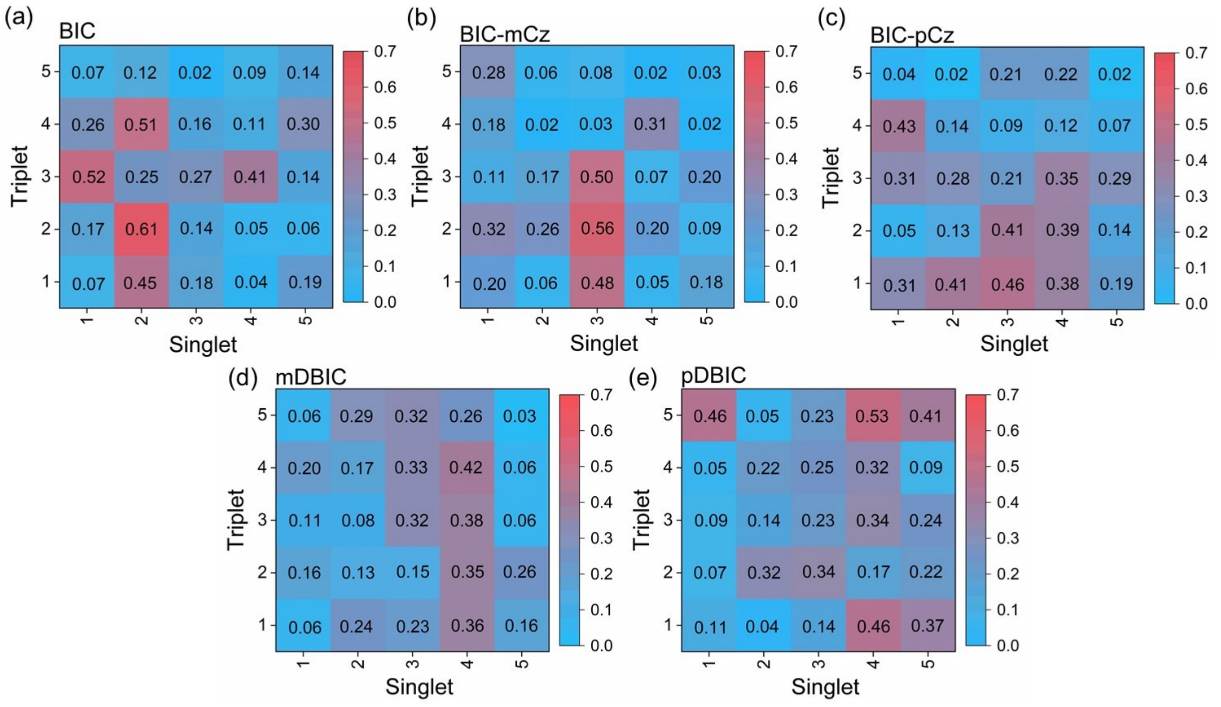

Figure S20. SOC heatmap of (a) **BIC**; (b) **BIC-mCz**; (c) **BIC-pCz**; (d) **mDBIC** and (e) **pDBIC**.

S4. Supplementary Transient Absorption Spectra

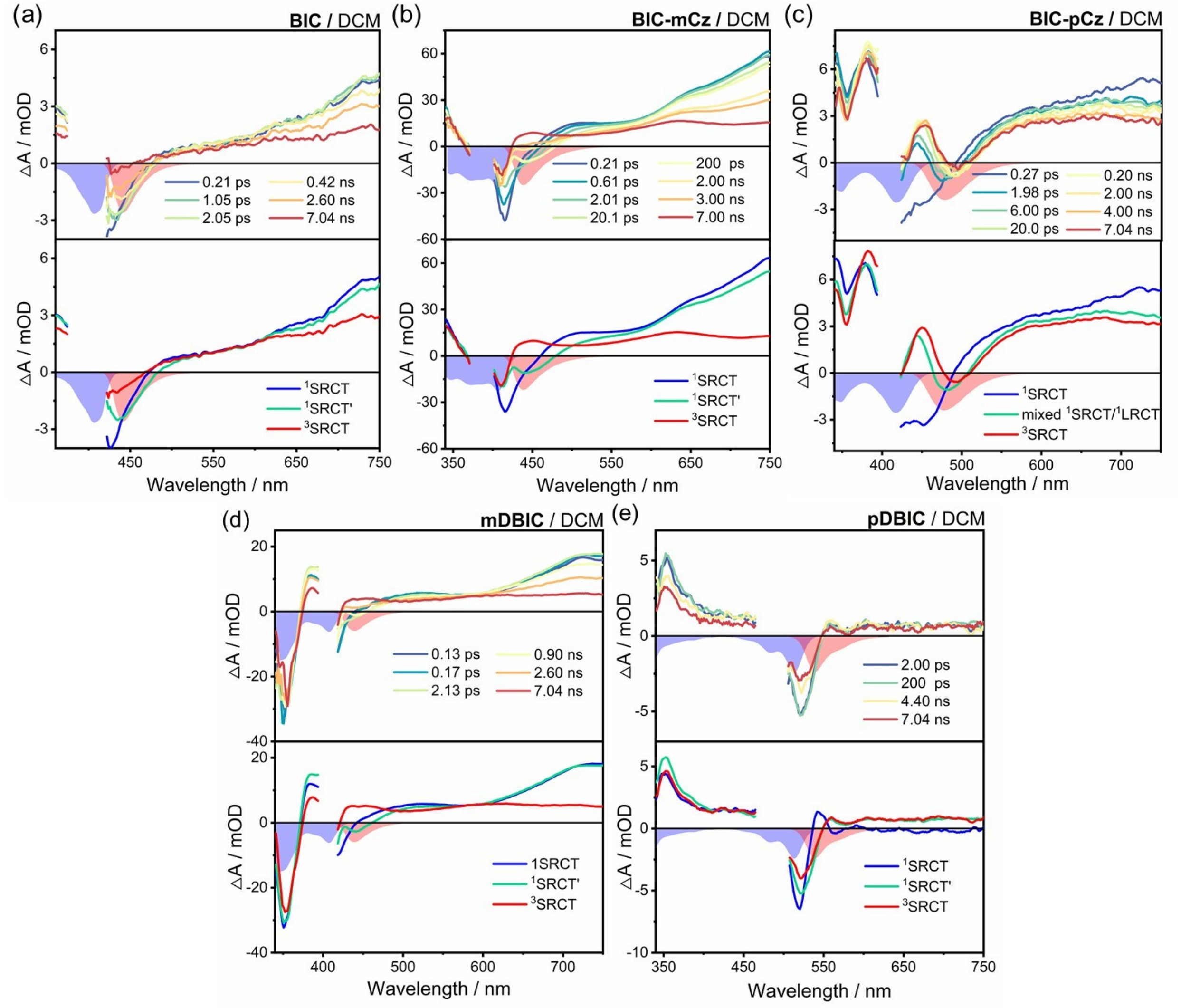

Figure S21. Time evolution of femtosecond TA spectra of the (a) **BIC** (λ_{ex} = 385 nm), (b) **BIC-mCz** (λ_{ex} = 385 nm), (c) **BIC-pCz** (λ_{ex} = 405 nm), (d) **mDBIC** (λ_{ex} = 385 nm) and (e) **pDBIC** (λ_{ex} = 480 nm) in DCM, respectively. The blue and red shaded areas represent the corresponding steady-state absorption and emission spectra, respectively. Evolution-associated different spectra (EADS) obtained from the global analysis based on a sequential model are shown in the lower panel of the corresponding TA spectra. The fitting time constants are shown in Table S12. SRCT: short-range charge-transfer state, SRCT′: structurally relaxed short-range charge-transfer state, LRCT: long-range charge-transfer state.

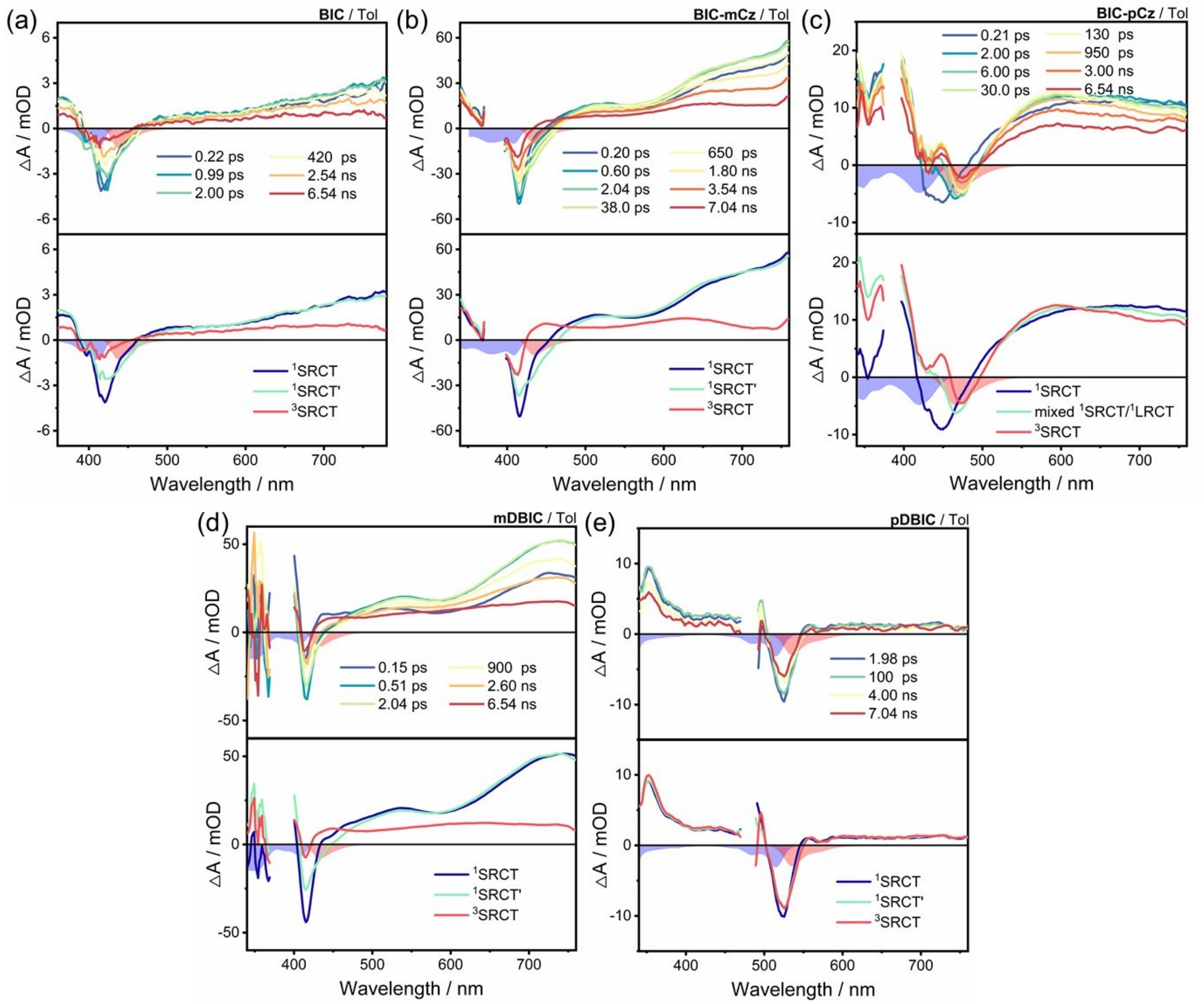

Figure S22. Time evolution of femtosecond TA spectra of the (a) **BIC** (λ_{ex} = 385 nm), (b) **BIC-mCz** (λ_{ex} = 385 nm), (c) **BIC-pCz** (λ_{ex} = 405 nm), (d) **mDBIC** (λ_{ex} = 385 nm) and (e) **pDBIC** (λ_{ex} = 480 nm) in toluene (Tol), respectively. The blue and red shaded areas represent the corresponding steady-state absorption and emission spectra, respectively. Evolution-associated different spectra (EADS) obtained from the global analysis based on a sequential model are shown in the lower panel of the corresponding TA spectra. The fitting time constants are shown in Table S12. SRCT: short-range charge-transfer state, SRCT′: structurally relaxed short-range charge-transfer state, LRCT: long-range charge-transfer state.

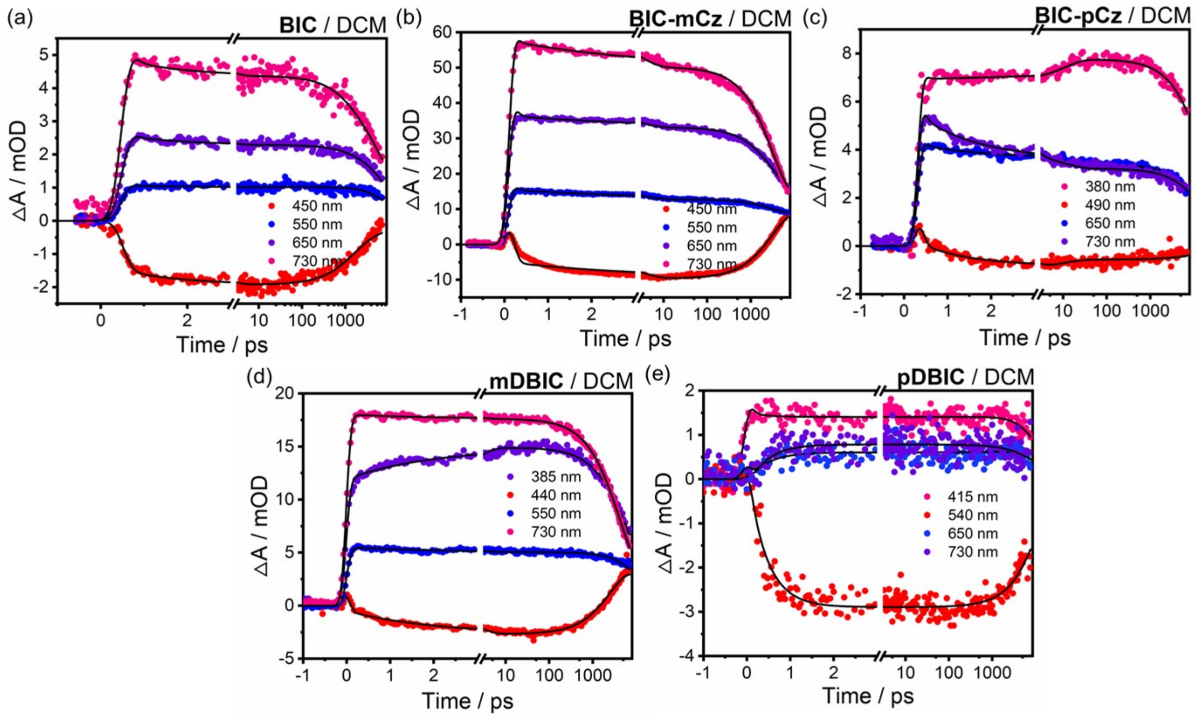

Figure S23. The fitting results of representative wavelength in the global target analysis of (a) **BIC**, (b) **BIC-mCz**, (c) **BIC-pCz**, (d) **mDBIC** and (e) **pDBIC** in dichloromethane (DCM), for showing the fitting quality.

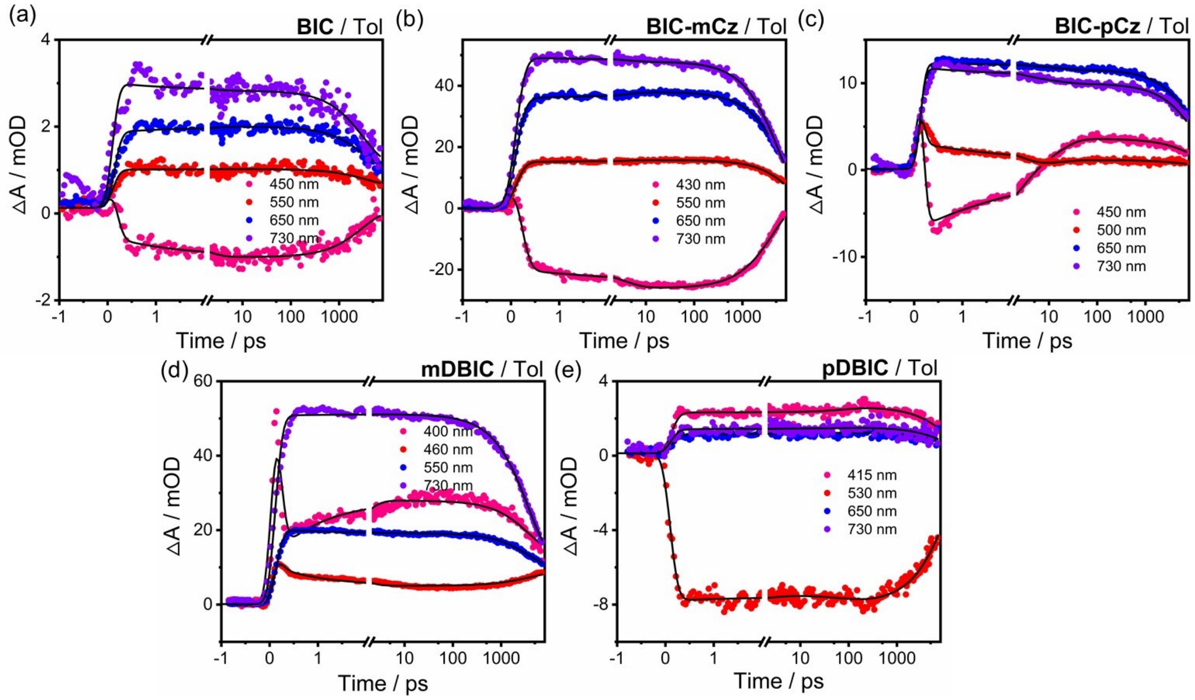

Figure S24. The fitting results of representative wavelength in the global target analysis of (a) **BIC**, (b) **BIC-mCz**, (c) **BIC-pCz**, (d) **mDBIC** and (e) **pDBIC** in toluene (Tol), for showing the fitting quality.

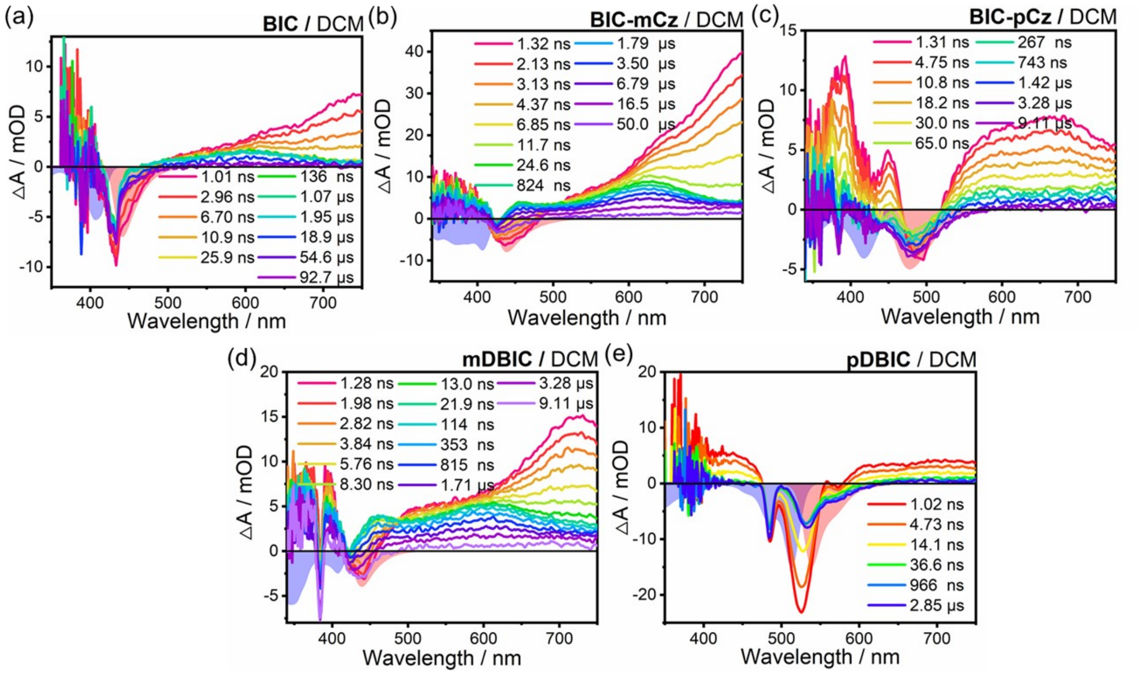

Figure S25. Time evolution of nanosecond TA spectra of the (a) **BIC** (λ_{ex} = 385 nm), (b) **BIC-mCz** (λ_{ex} = 385 nm), (c) **BIC-pCz** (λ_{ex} = 405 nm), (d) **mDBIC** (λ_{ex} = 385 nm) and (e) **pDBIC** (λ_{ex} = 480 nm) in DCM upon excitation at 385 and 480 nm under nitrogen conditions, respectively. The blue and red shaded areas represent the corresponding steady-state absorption and emission spectra, respectively.

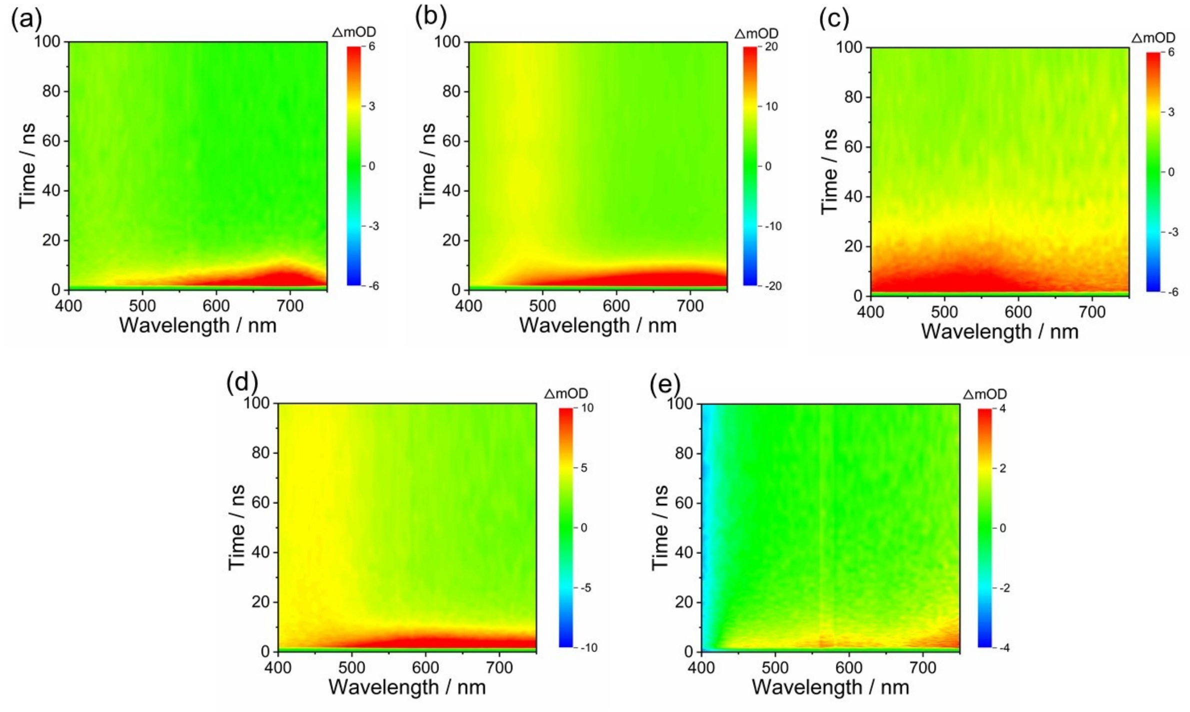

Figure S26.2D pseudo-color plots of ns-TA spectra of (a) **BIC**; (b) **BIC-Cz**; (c) B**IC-pCz**; (d) **mDBIC**; (e) **pDBIC** at nitrogen conditions.

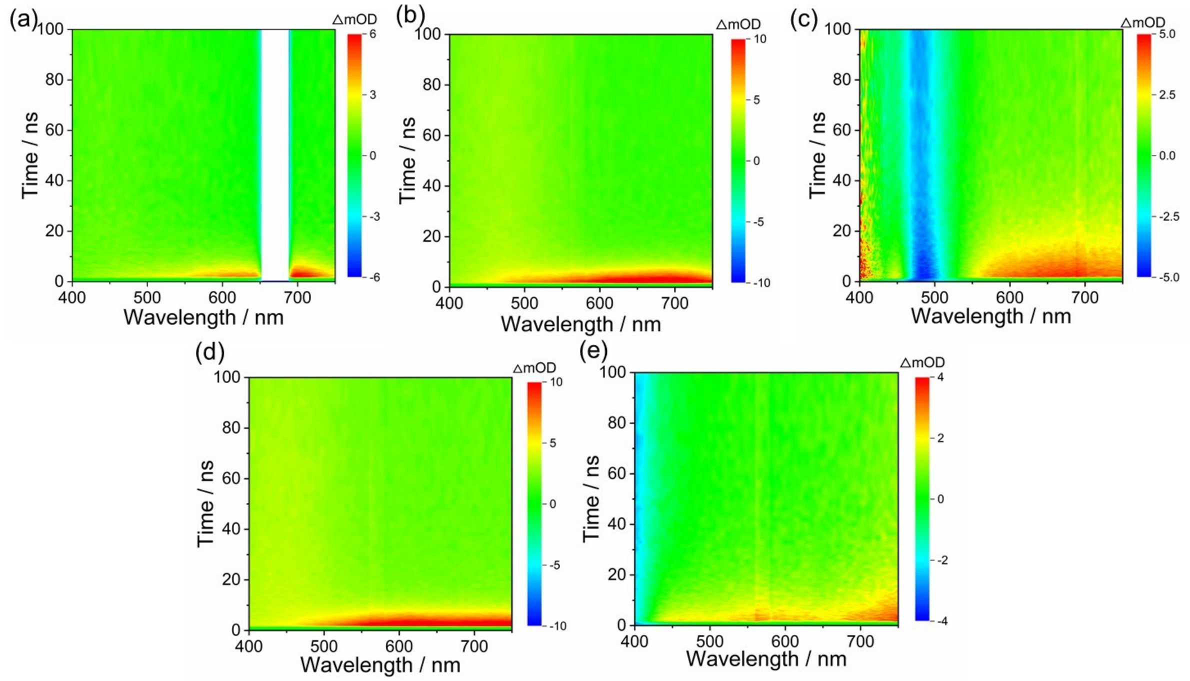

Figure S27.2D pseudo-color plots of ns-TA spectra of (a) **BIC**; (b) **BIC-Cz**; (c) B**IC-pCz**; (d) **mDBIC**; (e) **pDBIC** at air conditions.

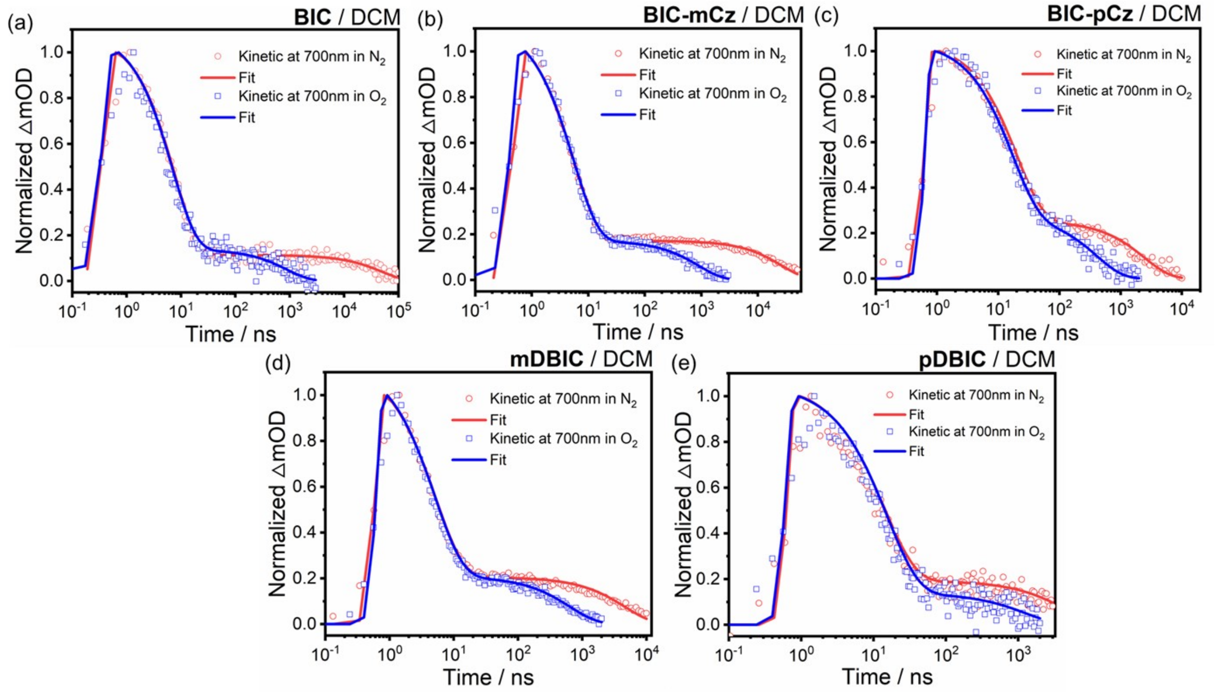

Figure S28. ns-TA spectra kinetic at 700 nm comparison of (a) **BIC**, (b) **BIC-mCz**, (c) **BIC-pCz**, (d) **mDBIC** and (e) **pDBIC** in DCM under nitrogen and oxygen condition.

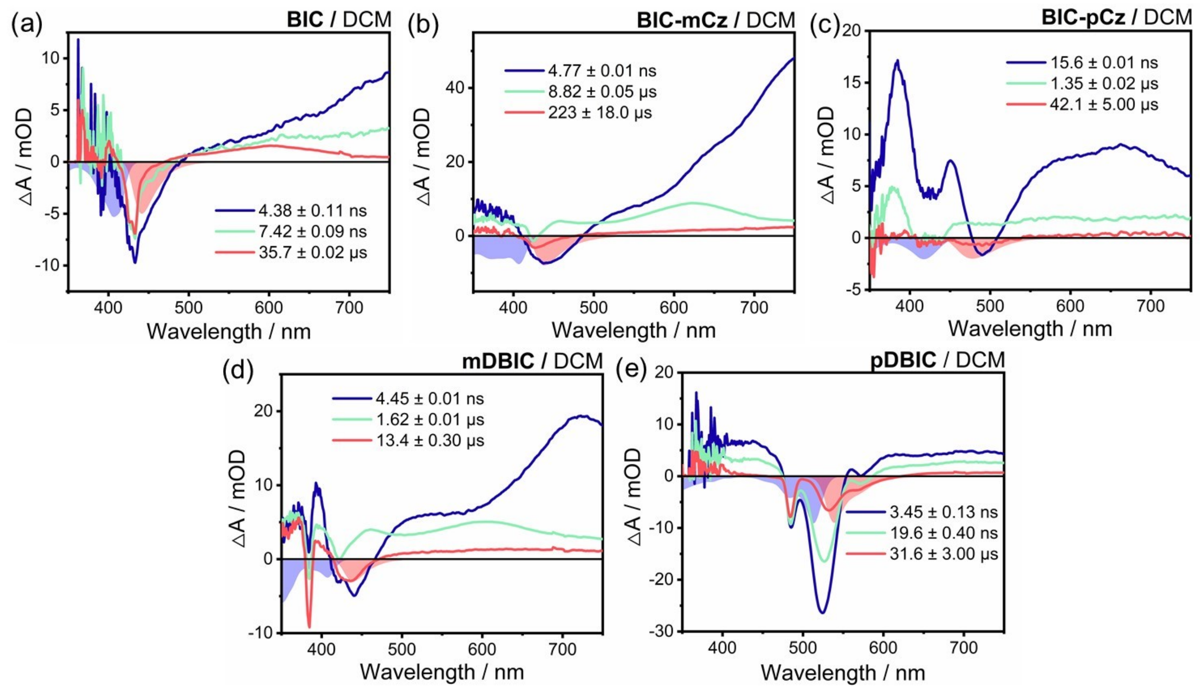

Figure S29. Evolution-associated different spectra (EADS) obtained from global analysis of ns-TA spectra for (a) **BIC**; (b) **BIC-mCz**; (c) **BIC-pCz**; (d) **mDBIC** and (e) **pDBIC** in DCM.

Table S12. Time Constants Estimated for Different Processes of **BIC**, **BIC-mCz**, **BIC-pCz**, **mDBIC** and **pDBIC** in Different Solvents Determined by fs-TA Measurements.

	solvent	τ_1 (ps)	τ_2 (ns)	τ_3 (ns)
**BIC**	Tol	1.47 ± 0.03	2.97 ± 0.06	long lived
	DCM	1.38 ± 0.02	1.42 ± 0.03	long lived
**BIC-mCz**	Tol	3.63 ± 0.03	4.90 ± 0.09	long lived
	DCM	3.05 ± 0.02	3.15 ± 0.03	long lived
**BIC-pCz**	Tol	1.05 ± 0.01	0.013 ± 0.00002	long lived
	DCM	1.24 ± 0.01	0.010 ± 0.00020	long lived
**mDBIC**	Tol	1.05 ± 0.02	3.71 ± 0.09	long lived
	DCM	1.99 ± 0.05	3.31 ± 0.09	long lived
**pDBIC**	Tol	2.59 ± 0.11	0.092 ± 0.0048	long lived
	DCM	0.35 ± 0.11	3.09 ± 0.54	long lived

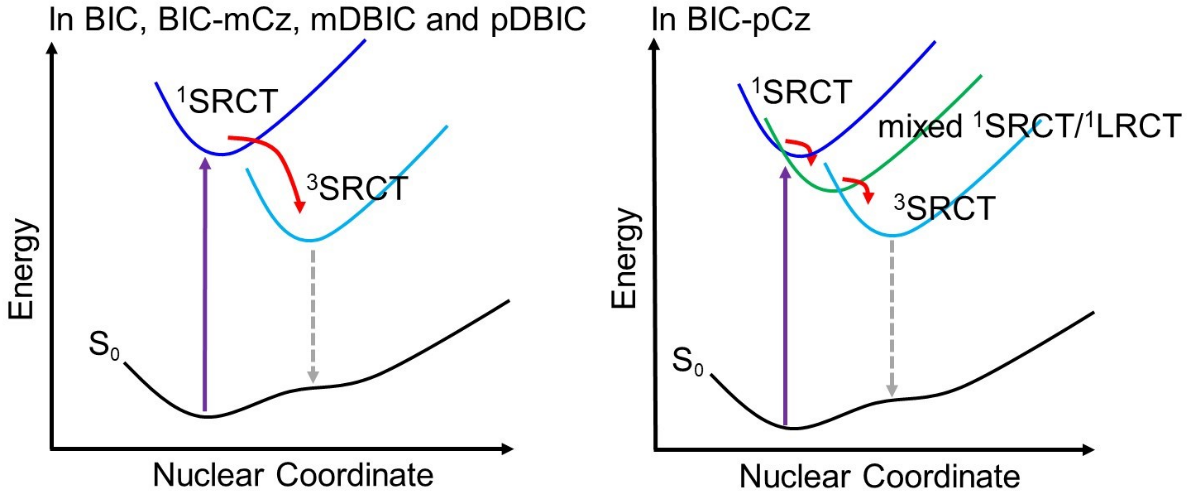

Figure S30. The proposed excited state relaxation mechanism of **BIC** derivatives.

S5. Supplementary References

(1) Wang, X.; Zhang, Y.; Dai, H.; Li, G.; Liu, M.; Meng, G.; Zeng, X.; Huang, T.; Wang, L.; Peng, Q.; Yang, D.; Ma, D.; Zhang, D.; Duan, L., Mesityl-Functionalized Multi-Resonance Organoboron Delayed Fluorescent Frameworks with Wide-Range Color Tunability for Narrowband OLEDs. *Angew. Chem. Int. Ed.* **2022,** *61* (38), e202206916.

(2) Snellenburg, J. J.; Laptenok, S.; Seger, R.; Mullen, K. M.; van Stokkum, I. H. M., Glotaran: A Java-Based Graphical User Interface for the R Package TIMP. *J. Stat. Soft.* **2012,** *49* (3), 1 - 22.

(3) van Stokkum, I. H. M.; Larsen, D. S.; van Grondelle, R., Global and target analysis of time-resolved spectra. *Biochim. Biophys. Acta* **2004,** *1657* (2), 82-104.

(4) Stephens, P. J.; Devlin, F. J.; Chabalowski, C. F.; Frisch, M. J., Ab Initio Calculation of Vibrational Absorption and Circular Dichroism Spectra Using Density Functional Force Fields. *J. Phys. Chem.* **1994,** *98* (45), 11623-11627.

(5) Zhao, Y.; Truhlar, D. G., The M06 suite of density functionals for main group thermochemistry, thermochemical kinetics, noncovalent interactions, excited states, and transition elements: two new functionals and systematic testing of four M06-class functionals and 12 other functionals. *Theor. Chem. Acc.* **2008,** *120* (1), 215-241.

(6) Frisch, M. J.; Trucks, G. W.; Schlegel, H. B.; Scuseria, G. E.; Robb, M. A.; Cheeseman, J. R.; Scalmani, G.; Barone, V.; Petersson, G. A.; Nakatsuji, H.; Li, X.; Caricato, M.; Marenich, A. V.; Bloino, J.; Janesko, B. G.; Gomperts, R.; Mennucci, B.; Hratchian, H. P.; Ortiz, J. V.; Izmaylov, A. F.; Sonnenberg, J. L.; Williams; Ding, F.; Lipparini, F.; Egidi, F.; Goings, J.; Peng, B.; Petrone, A.; Henderson, T.; Ranasinghe, D.; Zakrzewski, V. G.; Gao, J.; Rega, N.; Zheng, G.; Liang, W.; Hada, M.; Ehara, M.; Toyota, K.; Fukuda, R.; Hasegawa, J.; Ishida, M.; Nakajima, T.; Honda, Y.; Kitao, O.; Nakai, H.; Vreven, T.; Throssell, K.; Montgomery Jr., J. A.; Peralta, J. E.; Ogliaro, F.; Bearpark, M. J.; Heyd, J. J.; Brothers, E. N.; Kudin, K. N.; Staroverov, V. N.; Keith, T. A.; Kobayashi, R.; Normand, J.; Raghavachari, K.; Rendell, A. P.; Burant, J. C.; Iyengar, S. S.; Tomasi, J.; Cossi, M.; Millam, J. M.; Klene, M.; Adamo, C.; Cammi, R.; Ochterski, J. W.; Martin, R. L.; Morokuma, K.; Farkas, O.; Foresman, J. B.; Fox, D. J. *Gaussian 16 Rev. C.01*, Wallingford, CT, 2016.

(7) Lu, T.; Chen, F., Multiwfn: A multifunctional wavefunction analyzer. *J. Comput. Chem.* **2012,** *33* (5), 580-592.

(8) Humphrey, W.; Dalke, A.; Schulten, K., VMD: Visual molecular dynamics. *J. Mol. Graph.* **1996,** *14* (1), 33-38.

(9) Neese, F., Software update: The ORCA program system—Version 5.0. *WIREs. Comput. Mol. Sci.* **2022,** *12* (5), e1606.
